# Circulating microparticles: square the circle

**DOI:** 10.1186/1471-2121-14-23

**Published:** 2013-04-22

**Authors:** Natasha S Barteneva, Elizaveta Fasler-Kan, Michael Bernimoulin, Joel NH Stern, Eugeny D Ponomarev, Larry Duckett, Ivan A Vorobjev

**Affiliations:** 1 Program in Cellular and Molecular Medicine, Boston Children’s Hospital, D-249, 200 Longwood Avenue, Boston, MA 02115, USA; 2 Department of Pediatrics, Harvard Medical School, Boston, MA, USA; 3 Institute of Chemistry and Bioanalytics, University of Applied Sciences Northwestern Switzerland (FHNW), Muttenz, Switzerland; 4 Department of Biomedicine, University Hospital Basel, Basel, Switzerland; 5 Division of Hematology, University Hospital, Geneva, Switzerland; 6 Department of Developmental Biology, Harvard School of Dental Medicine, Boston, MA, USA; 7 Center for Neurologic Disease, Brigham and Women’s Hospital, Harvard Medical School, Boston, MA, USA; 8 Thematic Research Program for Neurodegeneration, Development and Repair, School for Biomedical Sciences, The Chinese University of Hong Kong, Shatin, NT, Hong Kong; 9 BD Biosciences Inc, San Jose, CA, USA; 10 A.N. Belozersky Institute for Physico-Chemical Biology and Department of Cell Biology, Biological Faculty, M.V. Lomonosov Moscow State University, Moscow, Russia

**Keywords:** Circulating, Microparticles, Exosomes, Microvesicles, Disease, Diagnostics, Therapy

## Abstract

**Background:**

The present review summarizes current knowledge about microparticles (MPs) and provides a systematic overview of last 20 years of research on circulating MPs, with particular focus on their clinical relevance.

**Results:**

MPs are a heterogeneous population of cell-derived vesicles, with sizes ranging between 50 and 1000 nm. MPs are capable of transferring peptides, proteins, lipid components, microRNA, mRNA, and DNA from one cell to another without direct cell-to-cell contact. Growing evidence suggests that MPs present in peripheral blood and body fluids contribute to the development and progression of cancer, and are of pathophysiological relevance for autoimmune, inflammatory, infectious, cardiovascular, hematological, and other diseases. MPs have large diagnostic potential as biomarkers; however, due to current technological limitations in purification of MPs and an absence of standardized methods of MP detection, challenges remain in validating the potential of MPs as a non-invasive and early diagnostic platform.

**Conclusions:**

Improvements in the effective deciphering of MP molecular signatures will be critical not only for diagnostics, but also for the evaluation of treatment regimens and predicting disease outcomes.

## Background

The present review summarizes information concerning microparticles (MPs), covering the clinical aspects of circulating MPs, recent advances and technological developments in this field.

## Implementation

Several recent reviews have concentrated on specific aspects of cellular vesicles biology, focusing primarily on exosomes (subset of cellular vesicles with size < 100 nm) and the mechanisms involved in cellular vesicles release and signaling [1-6]. This review focuses on another subset of cellular vesicles, i.e. microparticles (MPs). MPs are submicron vesicular fragments of cells that can be released by diverse eucaryotic and procaryotic cells and multicellular organisms under conditions of stress/injury [7-9]. Although novel methods to identify and characterize MPs have been developed in the last decade, classification of MPs, understanding of the molecular mechanisms of their release and biological function are still under intensive scrutiny [10-14]. The aims of this review article are to provide i) a systematic overview on circulating MP biology, and ii) a comprehensive description of the role of MPs in different diseases, based on the analysis of over 200 publications addressing changes in circulating MPs during pathological processes.

## Results and discussion

### MPs: attempts to define

MPs are described as a heterogeneous population of membrane-delimitated vesicles 50–1000 nm in size released from the cells in which they form and retaining certain antigens of their cells of origin [8,14,15]. MPs could be distinguished from other groups of cell-derived vesicles such as exosomes and apoptotic bodies. Exosomes are small vesicles (40–100 nm) that form through constitutive exocytosis of multivesicular endosomes [4,8], and often contain endocytic markers, such as tetraspannins and HSP73 [2,16]. MPs (also called “ectosomes”) form mostly by reverse budding and fission of the plasma membrane [17]. Because exosomes and MPs are often released concomitantly, differentiation of these two microvesicular species is difficult [18].

The size of MPs (50 to 1000 nm), their lipid composition, and their irregular shape and density are major parameters that separate them from exosomes (usually of diameter < 100 nm and lower density – 1.13-1.19 g/mL) and apoptotic bodies (much larger vesicles released at the final steps of apoptosis and normally 1000–3000 nm in size) [8,19]. This variance in reported size of MPs could occur due to limitations in the methods of the detection of MPs and differences in MP purification protocols, such as the anticoagulant used, centrifugation speed, filtration conditions, and type of storage used [20,21]. Besides, the majority of MPs express on their surface phosphatidylserine (PS) whereas PS is usually absent from exosomes’ surface [22]. In general, exosomes are smaller than MPs; however, reported sizes of MPs vary by publication, ranging from 50 nm to 1000–2000 nm (Additional file 1) and thus it is better to say that different research protocols allows one to enrich preparation with certain type of vesicles but not to separate them as a pure fraction. Current nomenclature of cell-derived vesicles was exhaustively presented recently [8], and we will follow it using terms *microparticle* and *microvesicle* as synonyms.

### Methods of MPs characterization

Isolation of MPs typically involves a combination of centrifugation and size-based filtration followed by characterization using flow cytometry, electron microscopy, Western blotting or proteomics. Isolation of MPs from the peripheral blood of patients or healthy controls starts with drawing blood into the tubes with different anticoagulants: sodium citrate, acid-citrate-dextrose, EDTA salt, or heparin. Sodium citrate is the most widely used anticoagulant [23]; however, blood collected with sodium citrate usually gives significantly lower levels of PS-positive MPs than blood collected in heparin [24]. Centrifugation is a critical step as well, since it can induce additional shedding of MPs from some cell types [24-26]. It is also possible that MPs can fuse during preparation, as MPs isolated by centrifugation are somewhat bigger than MPs in native MP-containing biological samples [27]. Haemolysis during sample preparation can significantly affect the amount of MPs isolated from plasma, as well as amounts of MP-related molecules like miRNA [28]. The size distributions of platelets (2–3 μm) and MPs (up to 2 μm) partially overlap, and current consensus indicates that the best way to remove contaminating platelets from MP preparations is via filtration. However, filtration of MPs should be used with caution, since this procedure can lead to fragmentation of larger MPs [29]. Finally, storage of purified MPs even at −80°C may further modify their characteristics [24,30].

Research focused on elucidating MP composition and functional activity is hampered by the complexity of the biological fluids where MPs are present and the small size of MPs [31]. Electron microscopy (EM) gives the diameter of individual MPs, but does not always provide quantitative data on the MP population - particularly when negative staining or cryoelectron microscopy are used. On ultrathin sections MPs appear as single, membrane-bounded vesicles with diameters ranging between 20–40 nm [32-35] and 300–700 nm [36-42], with the larger MPs exhibiting heterogeneous internal content. MPs as large as 1 μm in diameter were described using freeze-fracture and scanning EM [32-35]. Besides EM, atomic force microscopy and dynamic light scattering have been used for MP characterization [21,27,29,31].

The protein content of MPs is usually ascertained by Western blotting and proteomic approaches [43,44]. These assays require large numbers of MPs, limiting their utility for translational studies that require serum or other bodily fluids [45]. To date, only flow cytometry and microscopy methods have proved capable of providing specific information on the presence or absence of specific antigens in MPs derived from limited amounts of material. The application of different methods to exosome and MP research has been summarized by Van der Pol and coauthors [8,31,46], and in a number of recent publications [21-24,47,48].

**Figure 1:**
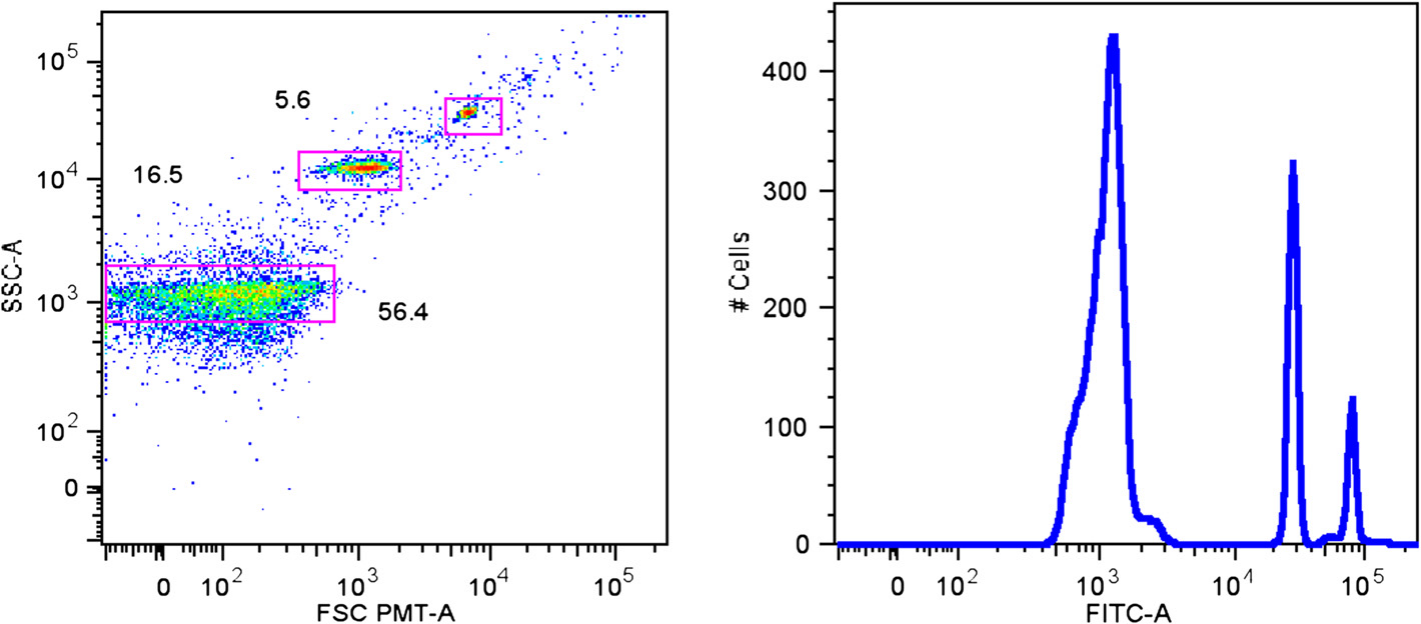
**Distribution of Dragon Green-conjugated beads in sizes of 190 nm, 510 nm, and 730 nm (images acquired with a SORP Aria 2 cytometer, Flow and Imaging Cytometry Resource, PCMM, Boston Children’s Hospital).** This figure was included in an advanced abstract as part of the Proceedings of the International Workshop on Applied Cytometry (2012).

**Figure 2:**
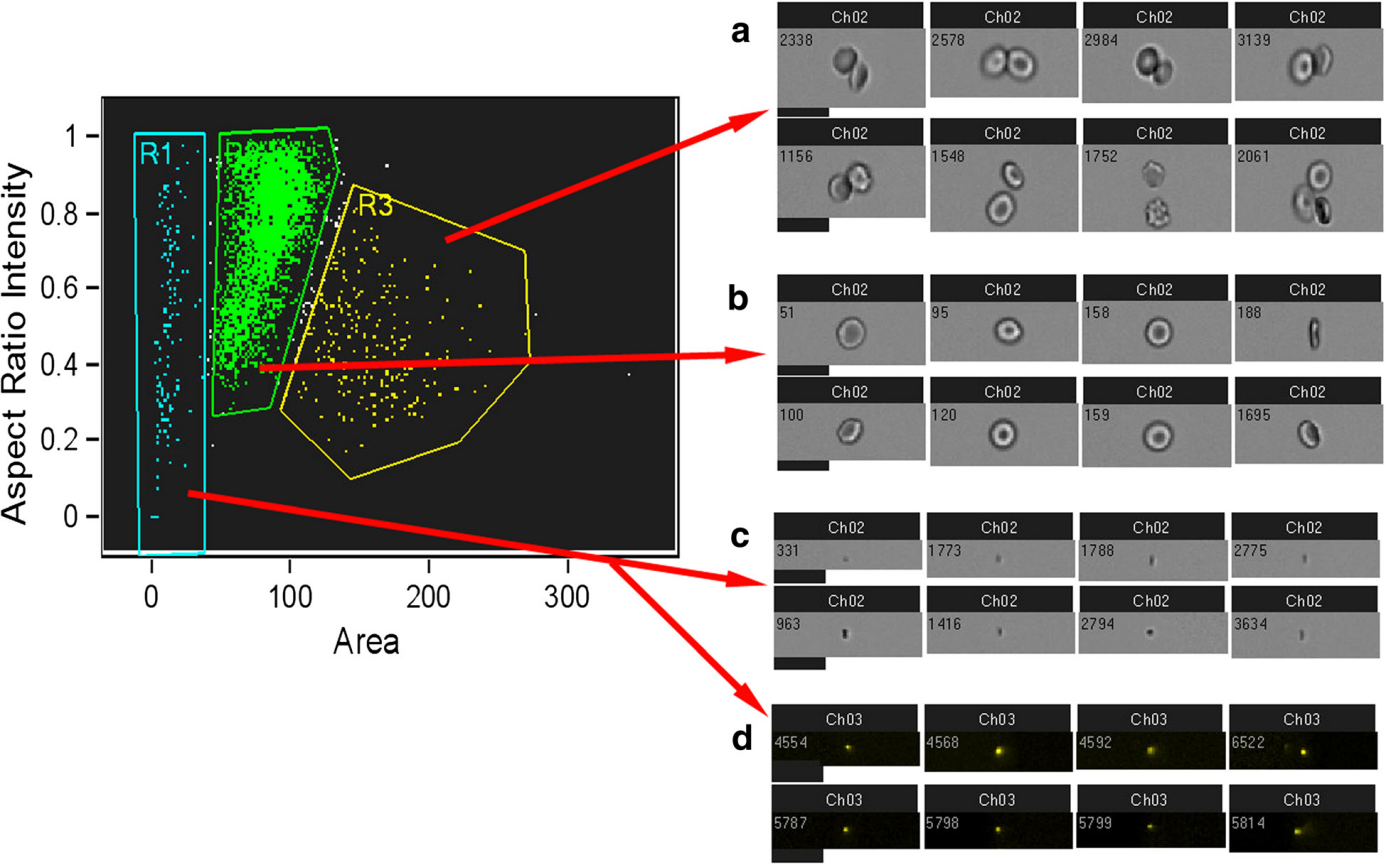
**MP images (erythrocyte-derived MPs) acquired with an Imagestream 100 (40x objective) (Amnis Inc, Seattle, USA).** A dotblot showing a mixture of erythrocytes and erythrocyte-derived microparticles (X-axis-brightfield area; Y-axis-brightfield aspect ratio of intensity). **a**.) Multiple erythrocytes (region R3 on dotblot); **b**.) Single erythrocytes (region R2 on dotblot); **c**.) Microparticles (brightfield) (region R1 on dotblot); **d**.) Microparticles stained with calcein AM (images from calcein AM-channel are particles taken from R1-region).

Current flow cytometry methods utilize both fluorescence probes and light scattering. Quantification of MPs by flow cytometry shows good correlation with the relative light scattering intensities determined by dynamic light scattering [49]. There are also indirect approaches for MP enumeration based on their functional activities [50,51]. However, conventional flow cytometry light scattering has size limitations and usually not able to detect microvesicles with diameters smaller than 300–400 nm as a separate fraction [31,52]. Particle size can be directly measured using impedance-based Coulter-type cytometers, but the sensitivity of this technology is also limited by 300–500 nm [31,52,53]. One other widely employed cytometric approach for the identification and characterization of MPs involves the use different sized beads as references [53,54]. However, the refractory index of polystyrene or other synthetic beads is higher than that of MPs, thus signals generated by MPs are very small. While conventional cytometers equipped with a photodiode for measuring forward light scatter have significant limitations in sensitivity for MP analysis, cytometers equipped with a photomultiplier in the forward scatter channel allow for better resolution of MP fractions (Figure 1, SORP FACSAria (BD Biosciences, San Jose, USA)). MPs can be directly stained with fluorescent antibodies and with fluorescent lipophilic dyes, both of which dramatically increase the ability of the cytometer to separate MPs from debris. For the best detection, MP staining for flow cytometry should include a lipid marker such as calcein AM, PKH67, or bio-maleimide [54-56], since staining MPs with only specific antibodies (AB) or annexin V can leave a significant percentage of MPs unstained or poorly stained and, as a result, lead to underestimation of MP levels. Recently, investigators have begun to use flow image cytometry for MP characterization (Figure 2). The advantages and disadvantages of commonly used methods for MP quantification and characterization are summarized in Table 1.

**Table 1:** Summary of some methods applied for MPs research

**Method**	**Quantification**	**Cell origin and/or function identification**	**MPs size distribution**	**Limitations**	**References**
Electron microscopy	Limited	Limited (only for single labeling by immunoelectron microscopy)	Yes, but might be subjective due to limited number of measurements	Artifacts due to specimen preparation for negative contrast (drying, application of contrasting solution etc.)	Hess et al., 1999; Distler et al., 2005; Lima et al., 2009; Witek et al., 2009; Porro et al., 2010; Duarte et al., 2012; Gercel-Taylor et al., 2012
Functional assays (procoagulant activity, thrombin generation tests, ELISA-based tests etc.)	Yes (bulk)	No	No	Only information on procoagulant or thrombin generating activity available	Leroyer et al., 2007; Tesselaar et al., 2007; Salzer et al., 2008; Manly et al., 2009; Van der Heyde et al., 2011
Atomic Force Microscopy	Limited	Limited (requires development of AB-coated surfaces)	Yes, but might be subjective due to limited number of measurements	Artifacts due to abundance of cell debri and plasma protein	Salzer et al., 2008; Yuana et al., 2010; Leong et al., 2011; Nantakomol et al., 2012
Light scattering techniques (nanoparticle tracking analysis, submicron particle analysis, dynamic light scattering)	Yes	No*	Yes	Artifacts due to abundance of cell debri and plasma protein – samples requires special purification	Lawrie et al., 2009; Xu et al., 2010; Gercel-Taylor et al., 2012
Western blotting	Semi-quantitative	Yes	No	Requires significant amount of starting material (> 10 μg of vesicular material)	Abid Hussein et al., 2005; Salzer et al., 2008; Sander et al., 2008; Bebawy et al., 2009; Bernimoulin et al., 2009; Gercel-Taylor et al., 2012
Mass-spectrometry	No	Yes, allows identification of multiple proteins	No	Requires significant amount of starting material	Sander et al., 2008; Mayr et al., 2009; Rood et al., 2010
Flow Cytometry	Yes	Yes, allows identification of multiple antigens	Limited	Limited; >300 nm particle range (conventional flow cytometry); presence of protein aggregates may lead to artifacts sensitivity depends on cytometer	Orozco, Lewis, 2010; Zwicker et al., 2010; Ayers et al., 2011; Yuana et al., 2011; van der Heyde et al., 2011
Flow imaging cytometry	Yes	Yes, allows quantification of multiple antigens	No	Limited for bright fluorescence MPs	Van der Heyde et al., 2011

*custom modified NTA system allows limited number of fluorescent measurements (Gercel-Taylor et al., 2012).

**References for Table 1 (Additional file 2).

### Origin of MPs

MPs have been identified in human plasma, peripheral blood, cord blood, urine, saliva and cerebrospinal fluid [45,57-62]. In addition, MPs have been found at different sites in lung disease patients, such as in the sputum from cystic fibrosis patients [39], and in bronchoalveolar lavage fluid (BALF) from patients with acute respiratory distress syndrome or hydrostatic pulmonary edema [63,64]. MPs have also been described in human atherosclerotic plaque [65-67], ascites, postoperative drainage fluid, and chyloid fluid [41], as well as in immunologically privileged sites such as vitreous eye liquid and synovial liquid [68-72]. Large body of evidence suggests that MPs are derived from all cellular types. The origin of MPs is critical because MPs with similar shapes and diameters yet derived from different cell types possess unique functional capabilities. Aleman et al. showed that MPs (100–300 nm in size) derived from monocytes had higher ability to support clot formation, making it more dense and stable compared to PMPs [73]. It has long been thought that the majority of MPs in the peripheral blood of a healthy person are released from platelets and endothelial cells [24,74]. However, it was recently suggested that CD61-positive MPs (currently called “PMPs”) originate directly from megakaryocytes [75,76]. Rank et al. showed that patients undergoing hematopoietic stem cell transplantation after total body irradiation (12 Gy) exhibit a rapid decline of the level of peripheral blood MPs, with CD61^+^ MPs disappearing faster than platelets and MPs expressing CD63 or P-selectin, leading the authors to conclude that at least a fraction of CD61^+^ MPs originate from megakaryocytes [77].

To characterize the cellular origin of MPs in peripheral blood, the most common approach is to stain MPs with fluorescently-labeled AB directed against antigens of parental cells (for example CD41, CD61 and platelet-activation marker CD62 for platelets; glycophorin for erythrocytes; CD45 for lymphocytes; CD14 for monocytes; and so on) and to perform subsequent analysis by flow cytometry. However, a large variety of CD markers have been used by different groups to characterize background and activation of MPs derived from endothelial cells (CD31, CD34, CD62E, CD51, CD105, CD144, CD146) versus platelets (CD41, CD41a, CD42a, CD42b, CD61, CD62P) may have led to inconsistency in the functional characterization of MPs populations (reviewed in [15]).

### Shedding (ectocytosis) and MP content

Though MP shedding is enhanced upon cell activation, constitutive ectocytosis is a permanent ongoing process *in vivo* for the majority of cells and significant levels of MPs originating from different cells can be always found in the plasma [78,79]. MPs contain a wide range of biomolecules: proteins (signal proteins and receptors, cytoskeleton and effector proteins), lipids, and nucleic acids, (e.g. microRNA, mRNA, and even DNA). MP surface protein content may be different from that of the plasma membrane of the cell of origin, as the incorporation of protein molecules into MPs can be a selective and modulated by agonist activators and/or microenvironments of the parental cells [54,80-84]. Depending on the stimulus, the protein content of MPs derived from the same cell lineage can vary. Jimenez et al. [85] demonstrated that endothelial cells release qualitatively and quantitatively distinct MPs in response to TNF-α (activation stimulus) and upon the induction of apoptosis by growth factor deprivation. In addition, several groups performing MP proteomic profile studies have found that characteristics of MPs isolated from peripheral blood depend on the type of stimulus used for their generation [54,86]. It has been shown that the density of β2-integrin and P-selectin is markedly enhanced in platelet-derived MPs (PMPs), whereas MPs from activated neutrophils are highly enriched in activated Mac-1 (10-fold enrichment) [87,88]. Moreover, the surface of PMPs is 50 to 100-fold more procoagulant than the surface of activated platelets [87]. It is likely that specific protein enrichment of MPs membrane is due, at least in part, to lateral re-organization of membrane lipids into cholesterol-rich lipid rafts during MP shedding [89,90]; however, the exact mechanisms involved in this process requires further investigation.

**Figure 3:**
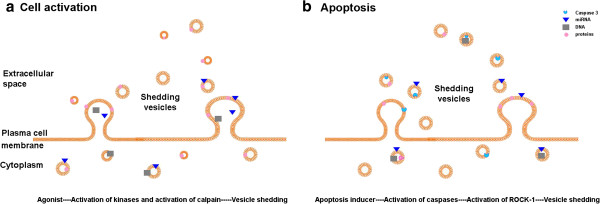
**Basic scheme of MP formation via reverse budding. A.** Activated cells release MPs in response to Ca^++^ agonists. Increased concentration of Ca^++^ alter the asymmetric PS distribution of the plasma membrane, activate kinases, inhibit phosphatases and activate calpain, which leads to reorganization of cytoskeleton and increased MPs production. **B.** MP formation during the early stages of apoptosis is associated with GTP-bound Rho proteins, which activate the ROCK-I kinase. This kinase is involved in cortical myosin-II contraction, detachment of the plasma membrane from the cytoskeleton, and release of MPs that have hijacked cytoplasmic components, nucleic acids, and membrane antigens.

Plasma membrane remodelling is a critical event during apoptosis and cell activation, and enzymes that regulate this process also regulate MP production [14]. The formation of MPs in response to activating stimuli is initiated by an agonist-mediated increase in intracellular calcium (Figure 3a), activation of kinases and inhibition of phosphatases, and calpain activation [14]. Activation of calcium-dependent scramblase (an ATP-independent transporter) and floppase (an exofacially-directed, ATP-dependent transporter) [91] results in exposure of PS on the outer leaflet of the plasma membrane [92]. Levels of PS exposure depend on the type of stimulation [85,93-95]. However, in some cases the processes of PS exposure and MP generation can be separated [96]. Particularly in endotoxemia and sickle cell disease formation of a large number of annexin-negative MPs was described [97,98]. Concomitant with the exposure of PS on the outer leaflets of MP membranes, calcium-sensitive enzymes such as calpain and gelsolin are activated, which promotes subsequent vesiculation [99]. In addition to the pathways decribed above, MP formation and trafficking can occur via ARF6-regulated endosomal pathways [100]. The exact mechanisms of lipid scrambling, PS exposure on the outer membrane leaflet, and ultimately MP formation, can differ between cell types [101,102]. In any case, PS on the surface of MPs is an important factor in mediating their functional activity: PS acts as a major prothrombotic and procoagulation signal, enhancing activation of coagulation proteins, TF, and platelet aggregation [103]. The functional role of PS-negative MPs is still a subject of debate, though elevated levels of circulating Annexin-negative MPs had been reported for initial phase of stroke, systemic lupus erythematosus (SLE) and some other diseases [104-107]. MPs can be captured by PS-binding molecules like T-cell immunoglobulin domain and mucin domain proteins, which are expressed on the surface of activated lymphocytes and phagocytes [108,109]. Formation and/or release of MPs can also be influenced by apoptotic signals [110] (Figure 3b). The shedding of MPs in response to apoptotic stimuli critically depends on the activation of Rho-associated kinase ROCK1 [111].

Several other enzymes possibly involved in MPs formation and activity include aminophospholipid translocase, and other members of the floppase family, as well as protein disulfide isomerase and acid sphingomyelinase [58,112-114]. Protein disulfide isomerase (PDI) – enzyme modulating flippase and floppase activities and regulating coagulation on endothelial cells [112] was shown to be a component of MPs released during tissue factor (TF)-dependent thrombosis [113]. Recently, Bianco et al. [114] demonstrated that activation of acid sphingomyelinase is necessary and sufficient for MP release by glial cells. As mentioned above, it is likely that lipid rafts are important participants in MP formation, since the depletion of plasma membrane cholesterol or raft disruption by methyl-cyclodextrin reduces MP release from a variety of cell types [89,115,116].

Enhanced release of MPs is associated with diverse stimuli including hormones, fatty acids, reactive oxygen species (e.g. hydrogen peroxide) [117], increased intracellular calcium levels [99]. Increased MP output is also driven by signals transduced through specific activating receptors, such as the purinergic receptor P2X on monocytes and neutrophils, thrombin receptors on platelets, and Toll-like receptor 4 (TLR4) on dendritic cells [118]. The level of MPs in human plasma can increase or decrease in response to different hormones, such as progesterone, estradiol, estrogen, insulin and others [119-121]. For example, low levels of estrogen in the blood are associated with increased microvesiculation and MP release [122]. Treatment with glucocorticoids significantly decreases the level of PMPs in peripheral blood in patients with polymyositis or dermatomyositis [123]. While insulin may promote MP release in certain cases, it has been found to reduce the procoagulant activity of MPs derived from lipopolysaccharide (LPS)-activated monocytes [124].

MPs also carry all types of nucleic acid molecules, including mRNA and DNA fragments [125,126]. Risitano et al. [127] demonstrated that platelet-derived mRNA could be transferred by MPs to monocytic and endothelial cell lines and undergo translation in the recipient cells. Improved ability to detect low copy numbers of small RNAs, including miRNA, has rapidly advanced the MP field, since these molecules has to be porotected from plasma nucleases and may be functional only when had been transferred by MPs internalized by target cells. Indeed, MPs from healthy donors contain miRNAs that have different functional activities [128], such as regulation of hemostasis [129]. Diehl and coauthors [130] assessed miRNA profiles of MPs derived from stimulated and non-stimulated endothelial cells (THP-1 and HUVECs) and found that miRNA profiles of MPs differed from those found in the stimulated or non-stimulated parental cells (some miRNAs upregulated while others down-regulated), suggesting a process of selective miRNA packaging into MPs. Specifically, MPs derived from stimulated THP-1 cells contained increased inflammatory miRNA and induced inflammation markers up-regulation in non-stimulated cells [130].

### Functional activities of MPs: interaction with homologous or heterologous cells

**Figure 4:**
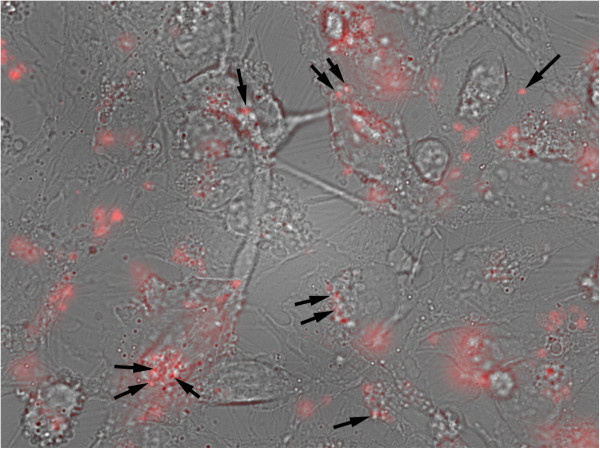
**An epifluorescence microscopy image shows that hCMEC/D3 cells have internalized small MPs (arrowheads), which had been purified from human glioma cells treated with TRAIL (100 units/ml) and stained with PKH-67 (Sigma, USA) before addition to the hCMEC/D3 cells.** MPs that are attached to the cell surface are out of focus (representative photo from Z-stack collection). Objective Plan Apo x60/1.4. Bar 5 μm.

As outlined above, MP production is a tightly regulated and selective process, suggesting that MPs may be important mediators of cell-to-cell communication. MPs can be internalized in a dose-dependent manner by macrophages, endothelial cells and other cell types (an example of MP internalization by hCMEC/D3 cells is shown in Figure 4). MP internalization can influence both functional and phenotypic characteristics of target cells. MPs may operate via surface interactions with receptor molecules on target cells or, more importantly, by directly transferring their contents, including RNA [130-133], bioactive lipids (for example platelet-activating factor (PAF) and PAF-like lipids), and proteins into the recipient cell [134,135].

The MPs express adhesion molecules on their surface, which may influence the probability of their capture by target cells and mediate MPs effects on cell behavior [136-138]. The cellular origin and site of release are essential factors in determining the functional activities of MPs. For example, MPs derived from red blood cells, but not from blood polymorphonuclears (PMNs) inhibit activation of macrophages by zymosan and LPS [139,140]. MPs participate in the release of insoluble proteins such as transmembrane receptors (CCR5, TF, EGFR, etc.) [90,141,142] and other surface molecules involved in immunomodulation [118,143,144]. The transfer of membrane-anchored receptors by MPs results in phenotypic alteration of the recipient cell, making it susceptible to different activating stimuli. For example, transfer of the chemokine receptor CCR5 by MPs to CCR5-deficient peripheral blood mononuclear cells makes them more sensitive to infection by CCR5-tropic HIV viruses [141]. Shuttling of the chemokine receptor CXCR4 by MPs contributes to HIV disease progression, since CXCR4 also serves as a co-receptor for some viruses [145]. Besides transferring receptor molecules, MPs may transfer chemokines, cytokines and growth factors to target cells [90,146]. For example, MPs transfer pro-apoptotic arachidonic acid between endothelial cells and circulating angiogenic cells [147], and constitute a main reservoir of blood-originated TF, the main activator of blood coagulation [142].

Lung-derived MPs have been shown to transfer mRNA to marrow cells [148], and MPs derived from endothelial progenitor cells have been reported to carry a wide range of mRNAs and to promote angiogenic activity and proliferation in quiescent endothelial cells [149]. Hemopoeitic stem cell-derived MPs contain mRNAs that contribute to the reprogramming of target cells [150]. Transfer of mRNAs to hepatocytes by liver stem cell-derived MPs induce proliferation and resistance to apoptosis [151]. Yuan et al. [152] demonstrated that miRNAs that are highly enriched within MPs are transferred to mTEC cells via MP internalization. miRNAs shuttled by MPs have been shown to downregulate the activity of proteins participating in cell proliferation and apoptosis such as cyclin D1, Bcl-2 and PTEN [153]. The most abundantly expressed miRNA in plasma MPs is miR-223, which participates in the maturation, proliferation and differentiation of myeloid and lymphoid cells [128]. MPs may also assist in the delivery to target cells of synthetic miRNAs [153].

A growing body of evidence supports an important role for MPs in the induction of apoptosis. MPs released at the early stages of apoptosis do not contain organelles and their size is smaller than 1 μm; however, they sediment at a lower acceleration than exosomes [110]. In contrast, so-called “apoptotic bodies”, which are released during the final stages of apoptosis, have a size of 1–4 μm, and often contain organelles [144]. Recently, Sarkar et al. [154] have demonstrated that monocyte-derived MPs induce death of target cells by delivering caspase-1. MPs from endothelial cells and platelets may also contain active executive caspase-3 [155-157]. Similarly, tumor-derived MPs serve as circulating cargoes for Fas ligand (FasL or CD95L), and therefore induce apoptosis in lymphoid target cells harboring the Fas receptor [158,159]. In addition to FasL, MPs and exosomes from different human tumors (melanoma, head, neck, ovary, colorectal and other cancers) may carry other proapoptotic molecules, such as TRAIL [143,159-161].

### Circulating MPs

The level of circulating MPs depends on the balance between their rates of formation and clearance. Clearance of MPs occurs through several main mechanisms. The major one is degradation due to the action of phospholipases and proteases [162]. Other potential routes of MP clearance include: (i) opsonization with subsequent phagocytosis; (ii) uptake of MPs from the circulation by liver Kupffer cells in a PS-dependent manner [163]; (iii) phagocytosis of MPs by splenocytes [164]; and (iv) uptake of MPs by the lung macrophages [165]. In a rat model, both the spleen and liver were found to participate in the clearance of MPs labeled with radioactive ^51^Cr, with only 12% of injected erythrocyte-derived microvesicles retained in the plasma after 60 min [166]. However, recent studies suggest that survival of PS^+^ MPs in human blood is rather long: the half-life of Annexin V^+^-MPs measured upon transfusion of apheresis platelet concentrates is approximately 5.8 hours and for CD61^+^ MPs it is 5.3 hours [167]. MP size is also an important factor in their clearance – strong inverse correlation between IgM-mediated clearance half-time and particle size of MPs by macrophages was determined [168]. On opposite, Al-Faraj et al. [169] demonstrated rapid clearance (within 5 min) of iron-labeled MPs by time-lapse molecular imaging using mouse model. However, it should be taken into account that labeling of such a fragile thing as MPs *ex vivo* may change clearance characteristics and kinetics.

While low MP concentrations can be detected in the blood and body fluids of healthy subjects [170-172] (summarized at Table 2), increased concentrations of MPs in the blood of patients with different pathological states supports the notion that MPs play a role in numerous diseases, including different cancers (Table 3), infectious diseases, autoimmune diseases, thromboembolic events and others (Table 4). However, most of these studies are observational and the possible role of MPs as prognostic biomarkers in stratification of disease risk groups is only starting to be addressed. There have been very few prospective studies aimed at evaluating whether there is an association between the quantities of a certain subtype of MP (endothelial, erythrocyte or other cell-derived MPs) and the outcome of diseases or therapeutic procedures [173-175]. Increased MP levels in pathological disorders such as intracerebral hemorrhage, endotoxemia, hepatitis C and others are generally associated with adverse outcomes (Additional file 3), and high levels of MPs associated with these disorders could, at least partly, be implicated in the vascular complications of these diseases. However, although increased levels of circulating MPs have been associated with various autoimmune diseases (SLE, rheumatoid arthritis, systemic sclerosis), facile correlation of MP quantity and adverse outcomes is complicated by the fact that plasma MP levels appear to increase to lower levels in patients with more severe disease [176]. Thus, the factors regulating MP release during desease progression are complex and yet remain to be evaluated. In this regard, it is important to consider the effect of pharmacological agents on circulating MP levels and their composition (summarized in the Additional file 4). Most of these studies have demonstrated that beneficial treatment of disease lowers circulating MP levels. For example, treatment of multiple sclerosis (MS) with interferon-β1 decreased the amount of circulating CD31^+^ endothelial MPs in plasma [177]. Similar results were obtained by Lowery-Nordberg et al. [178]. These data suggest that the quantity of specific MPs in the circulation may be used as a surrogate marker for interferon therapy responsiveness.

**Table 2:** MPs levels in the plasma of healthy controls

**Disease**	**MPs plasma levels**	**Reference**
Cord blood	Elevated MPs levels or activity comparing with mother’s plasma	Uszynski et al., 2011; Schweintzger et al., 2010; 2011
Healthy smokers	Elevated EMPs levels;	Gordon et al., 2011; Grant et al., 2011
	Diminished MP levels	
Healthy donors	MP levels	Berckmans et al., 2001; Bretelle et al., 2003
Normal pregnancy	Elevated MPs levels	Bretelle et al., 2003
Strenuous physical exercise	Elevated PMPs and PMN-MPs	Chaar et al., 2011
Gender	Elevated CD61^+^ MPs in men; no difference	Caby et al., 2005; Toth et al., 2007; Grant et al., 2011
Climacteric	Lowered PMPs levels, no impact on EMPs levels	Rank et al., 2012
Age (<18 years)	Elevated MPs levels	Proulle et al., 2005
Age (geriartric patients)	Decrease EMPs, altered MPs response to infection	Forest et al., 2010
High-fat meal	Elevated cycling blunts of CD18^+^ and CD11a^+^ MMPs and EMPs levels	Strohacker et al., 2012
Obesity	Elevated MPs levels; elevated CD144^+^EMPs	Goichot et al., 2006; Esposito et al., 2006; Gunduz et al., 2012
Endotoxemia (*E.coli* LPS) in healthy volunteers	Elevated TF^+^ MPs	Aras et al., 2004; Woei-A-Jin et al., 2012^*^

*References for Table 2 (Additional file 5).

**Table 3:** MPs levels in the plasma and body fluids of patients with cancer

**Disease**	**MPs plasma levels**	**Reference**
Acute myeloid leukemia	Elevated MPs levels; decreased during chemotherapy and increased during remission;elevated CXCR4^+^- MPs; elevated PMPs and myeloblast-derived MPs	Kalinkovich et al., 2006; Szczepanski et al., 2011; Van Aalderen et al., 2011
Acute lymphoid leukemia	MPs in bone marrow aspirate	Savasan et al., 2004
Acute promyelocytic leukemia	Elevated CD33^+^TF^+^ MPs	Ma et al., 2013
B-cell chronic lymphoid leukemia	Elevated MPs levels	Ghosh et al., 2009
Bladder cancer	Elevated MPs containing EGFR-associated proteins	Smalley et al., 2008
Brain cancer	TF^+^ MPs not elevated	Thaler et al., 2012
Breast cancer (getting endocrine therapy)	Elevated MPs levels;	Liebhardt et al., 2010; Trappenburg et al., 2011
	Elevated annexin V^+^, CEA^-^, BCRP^-^, HSP27^+^ subpopulations of MPs	
Breast cancer (metastatic)	Elevated TF^+^ MPs levels;	Tesselaar et al., 2007; Toth et al., 2008; Liebhardt et al., 2010
	Elevated PMPs and annexin V^+^-MPs; increased annexin V^+^, CD66^+^, BCRP1^+^ and HSP27^+^ MPs	
Colorectal cancer	Elevated TF^+^ MPs levels	Hron et al., 2007
Gastric cancer	Elevated MPs and PMPs levels	Kim et al., 2003; Baran et al., 2010
Glioblastoma multiforme	Elevated procoagulant MPs	Sartori et al., 2011
Gynecological cancer	MPs levels are not elevated	Zahra et al., 2011
Hepatocellular carcinoma	Elevated MPs levels	Brodsky et al., 2008
Lung cancer	Elevated MPs levels	Kanazawa et al., 2003
Non-small cell lung cancer	Elevated AnnexinV^+^-MPs	Fleitas et al., 2012
Melanoma	Elevated MPs levels	Lima et al., 2011
Multiple myeloma	Elevated MPs levels	Auwerda et al., 2011
Ovarian cancer	Elevated MPs levels; elevated CD63^+^ MPs comparing with benign ovarian tumors; Elevated EpCam + MPs in ascites at advanced stage	Ginestra et al., 1999; Taylor et al., 2002; Taylor, Gercel-Taylor, 2008; Rank et al., 2012; Press et al., 2012
Ovarian cancer (ascites)	Elevated epithelial cell adhesion molecule-positive MPs at advanced stages	Press et al., 2012
Pancreas cancer	Elevated TF^+^ MPs	Thaler et al., 2012
Prostate cancer	Elevated TF^+^ MPs; elevated MPs levels	Haubold et al., 2009; Coumans et al., 2010
Different tumor types	Elevated procoagulant MPs levels	Manly et al., 2010; Thaler et al., 2011
Cancer with thromboembolic complications	Elevated MPs levels	Zwicker JI et al., 2009
Tumor surgery (tumor mass removal)	MPs decreased	Zwicker et al., 2009; Sartori et al., 2011

*references for Table 3 (Additional file 6).

**Table 4:** MPs levels in the plasma and body fluids of patients with different disorders

**Disease**	**MPs plasma levels**	**Reference**
**AUTOIMMUNE DISEASES**		
Ankylosing spondylitis	No differences between patient and control groups in EMPs and PMPs levels	Sari et al., 2012
Anti-phospholipid syndrome	Elevated MPs levels; TF^+^ EMPs, monocyte-derived MPs	Joseph et al., 2001; Dignat-George et al., 2004; Jy et al., 2007; Vikerfors et al., 2012
Arthritis	Elevated MPs levels	Berckmans et al., 2002; Boilard et al., 2010
Acute inflammatory bowel disease	Elevated MPs levels; elevated TF^+^ MPs	Andoh et al., 2005; Palkovits et al., 2012
Behcet’s disease (systemic vasculitis)	CD62^+^-MPs levels elevated	Macey et al., 2011
Cirrhosis	Elevated CD31^+^/41^-^; CD11a^+^; CD4^+^; CD235a^+^; cytokeratin 18^+^ MPs	Rautou et al., 2012
Crohn’s disease	Elevated MPs levels comparing with normal and ulcerative colitis	Chamouard et al., 2005
Diabetes mellitus	Different patterns of MPs, PMPs na MMPs levels and also differences from diabetes type II pattern	Diamant et al., 2002; Sabatier et al., 2002; Shouzu et al., 2004; Ogata et al., 2005 Tramontano et al., 2010
Diabetic retinopathy	Increased vitreous shedding of MPs, endothelial,platelet, photoreceptor, and microglial origin	Ogata et al., 2005; 2006; Chahed et al., 2010
Diabetes type II (Diabetes mellitus)	Elevated MPs levels;	Nomura et al., 1995; Sabatier et al., 2002; Nomura et al., 2004b; Tan et al., 2005; Jung et al., 2009a; Koga et al., 2005; 2006; Leroyer et al., 2008; Nomura, 2009; Nomura et al. 2009; Bernard et al., 2009; Tsimerman et al., 2011; Nomura et al., 2011
	AnnexinV++ MPs elevated	
Kawasaki disease	Elevated MPs levels, especially EC and T-cells derived	Guiducci et al., 2011; Tan et al., 2012
Mixed connective tissue disease	Elevated PMPs levels	Oyabu et al., 2011
Multiple sclerosis	Elevated MPs and PMPs levels	Larkin, 2001; Minagar et al., 2001; Jy et al., 2004; Jimenez et al., 2005; Sheremata et al., 2006; 2008
Polymyositis/dermatomyositis	Elevated MPs and B-lymphocyte–derived MPs levels	Shirafuji et al., 2009; Baka et al., 2010
Psoriasis	Elevated PMPs levels	Tamagawa-Mineoka et al., 2010; Pelletier et al., 2011
Rheumatoid arthritis	Different patterns of MPs levels in plasma; increased PMPs expressing activating markers; increased MPs in synovial fluid; increased MPs exposing complement components (C1q, serum amyloid-P)	Joseph et al., 2001; Knijff-Dutmer et al., 2002; Berckmans et al., 2002; Biro et al., 2007; Sellam et al., 2009; Messer et al., 2009; Umekita et al., 2009; van Eijk et al., 2010
Sjorgen syndrome	Elevated MPs, PMPs, leukocyte-derived MPs levels	Sellam et al., 2009
Systemic lupus erythematosus	Elevated MPs levels; PMPs levels;	Combes et al., 1999; Joseph et al., 2001; Nagahama et al., 2001; Pereira et al., 2006; Sellam et al., 2009; Antwi-Baffour et al., 2010; Nielsen et al., 2011; 2012;
	Elevated levels of Annexin V-negative MPs;	
	Elevated annexin V^+^ CD31^+^ EMPs; elevated levels of MPs with increased loads of IgG, IgM and C1q	
Systemic sclerosis	Elevated MPs and PMPs levels	Guiducci et al., 2008; Nomura et al., 2008; Oyabu et al., 2011
Vasculitis	Elevated MPs levels	Brogan et al., 2004; Daniel et al., 2006; Erdbruegger et al., 2008
**BLOOD DISORDERS**		
**Aplastic anemia**	Elevated procoagulant MPs	Hugel et al., 1999
Beta-thallasemia	Elevated MPs levels; elevated annexin V^+^ MPs from plathelets and red blood cells	Pattanapanyasat et al., 2004; 2007; Habib et al., 2008; Chaichompoo et al., 2012
Disseminated intravascular coagulation (DIC)	Elevated MPs	Rahman et al., 2011
Essential thrombocytemia	Elevated PMPs and EMPs levels	Trappenburg et al., 2009
Haemophilia	Elevated MPs levels	Proulle et al., 2005
Henoch-Schönlein purpura (HSP)	Elevated EMPs levels	Dursun et al., 2010
Immune thrombocytopenic purpura (ITP)	Elevated MPs levels in acute phase and decreased in chronic phase; increased Er-Mps and PMPs levels	Jy, 1992;Tantawy et al., 2009; Sewify et al., 2013
Paroxysmal nocturnal hemoglobinuria	Elevated MPs and EMPs levels	Hugel et al., 1999; Liebman, Feinsten, 2003; Simak et al., 2004; Helley et al., 2010
Scott’s syndrome, Castaman syndrome, Glanzmann thromboasthenia (bleeding disorders)	MPs deficiency	Sims et al., 1989; Gemmel et al., 1993; Castaman et al., 1996; Toti et al., 1996
Sickle cell anemia	Elevated MPs levels; increased annexin V and PS-MPs levels, increased TF^+^-MPs; elevated Er-MPs	Shet et al., 2003; van Tits et al., 2009; van Beers et al., 2009; Gerotziafas et al., 2012
Thrombotic thrombocytopenic purpura	Elevated MPs and PMPs levels	Galli et al., 1996; Jimenez et al., 2001
**CARDIOVASCULAR DISEASES**		
Acute coronary syndrome	Elevated EMPs levels;	Bernal-Mizrahi et al., 2003; Biassuci et al., 2012
	Elevated Annexin V^+^, EMPs and PMPs levels	
Acute pulmonary embolism	PMPs elevated	Bal et al., 2010
Arterial erectile dysfunction	Elevated EMPs levels	La Vignera et al., 2012; Condorelli et al., 2012
Cardiomyopathy	Elevated MPs, MMPs levels; decreased endothelial MPs levels	Walenta et al., 2012
Cardiopulmonary resuscitation	Elevated Annexin V^+^-MPs	Fink et al., 2011
Cerebrovascular accidents	Elevated MPs levels; EMPs, PMPs elevated in patients with subarachnoid hemorrhage and acute cerebral infarction	Lee et al., 1993; Jung et al., 2009b; Lackner et al., 2010; Kuriyama et al., 2010
Chronic venous unsufficiency	Elevated EMPs and PMPs levels	Georgescu et al., 2009
Coronary artery disease	CD31^+^, Annexin V^+^ MPs increased	Werner et al., 2006; Amabile et al., 2011
Hypertension	Elevated eMPs	Preston et al., 2003; Huang et al., 2010
Myocardial infarction	Elevated MPs and PMPs levels	Stepien et al., 2012
Non-valvular atrial fibrillation	PMPs elevated	Choudhury et al., 2007
Pulmonary hypertension	Elevated CD62^+^ EMPs, leukocyte-derived MPs	Amabile et al., 2008, 2009; Bakouboula et al., 2008
Thromboangiitis obliterans (Buerger’s disease)	Elevated MPs during exacerebration	Damige et al., 2010
Valvular atrial fibrillation	CD41^+^ PMPs elevated	Azzam, Zagloul, 2009
Vasculites associated with anti-neutrophil antibodies (Wegener’s granulomatosis; Churg-Strauss syndrome; microscopic polyangiitis)	PMPs, NMPs and EMPs elevated	Brogan et al., 2004; Daniel et al., 2006; Erdbruegger et al., 2008; Kuempers et al., 2008
Deep vein thrombosis	MPs levels are not increased	Steppich et al., 2011
Venous thromboembolism	Elevated EMPs	Chirinos et al., 2005
Unstable angina, Cardiovascular disease, arteriosclerosis obliterans, atherosclerosis, ischemic stroke	Elevated MPs and PMPs levels;	Singh N, 1995; Mallat et al., 2001; Nomura et al., 2004a; Dymicka-Piekarska et al., 2005; Zielinska et al., 2005; Morel et al., 2005; Simak et al., 2006; Michelsen et al., 2009; Kim et al., 2012
	Elevated CD105^+^ (mesenchymal stem cell marker) after stroke, especially extensive ischemic stroke	
**INFECTIOUS DISEASES**		
Hepatitis C	Elevated T-cell MPs levels correlated with severity of disease	Kornek et al., 2011, 2012
Hepatitis C with cirrhosis	Elevated MPs levels comparing with HepC; elevated MPs from CD4+ and CD8^+^ T-cells	Brodsky et al., 2008
HIV	Elevated MPs and EMPs levels; upregulation TF and P-selectin	Gris et al., 1996; Holme et al., 1998; Corrales-Medina et al., 2010; da Silva et al., 2011; Mayne et al., 2011
Hemolytic uremic syndrome (enterohemorrhagic *Escherichia coli* infection)	Elevated PMPs and MMPs levels	Stahl et al., 2009; 2011
*Plasmodium falciparum* and *P. vivax* infections	Elevated MPs levels, Er-MPs levels	Combes, 2004; 2005; Campos et al., 2010; Pankoui Mfonkeu et al., 2010; Nantakomol et al., 2011
Sepsis (menningococcal)	Elevated procoagulant MPs levels	Niewland et al., 2000
Sepsis (*Streptococcus pyogenes)*	Elevated PS^+^-MPs levels	Oehmcke et al., 2011
Sepsis (pneumococcus, enterococcus, staphylococcus-associated)	Elevated endothelial protein C-receptor^+^-MPs	Perez-Casal et al., 2011
Sepsis and trauma	Different patterns of MPs levels	Joop et al., 2001; Ogura et al., 2001; Fujimi et al., 2003; Morel et al., 2008; Mostefai et al., 2008; Park et al., 2012
Sepsis (*Candida albicans*)	Elevated CD42a^+^ and PAC1^+^ PMPs	Woth et al., 2012
Shiga-toxin induced haemolytic	Elevated MPs (platelets, monocytes, granulocytes)	Ge et al., 2012
uraemic syndrome (HUS)		
Systemic Inflammatory Response syndrome (SIRS)	Elevated MPs levels	Ogura et al., 2004
**FEMALE DISORDERS**		
Polycystic ovary syndrome (PCOS)	Elevated pMPs levels in women with PCOS and hyperandrogenemia	Koiou et al., 2011; 2013
Pre-eclampsia and eclampsia	Different patterns of MPs levels compared with normal pregnancies; endothelial CD41^-^ MPs elevated; CD62^+^ MPs elevated; MMPs and CD8^+^ and granulocyte-derived MPs elevated	VanWijk et al., 2002; Goswami et al., 2006; Lok et al., 2008; 2009; Macey et al., 2010; Reyna-Villasmil et al., 2011; Alijotas-Reig et al., 2012
Pathological pregnancies	PMPs levels decreased comparing with normal pregnancies	Bretelle et al., 2003; Carp et al., 2004
Postmenopausal women taking hormone replacement therapy	Elevated MPs from platelets/megakaryocytes (CD61^+^)	Rank et al., 2012
**KIDNEY DISORDERS**		
Chronic renal failure	CD144^+^ and CD146^+^ EMPs elevated	Amabile et al., 2005; Faure et al., 2006
Different nephropathies (nephrosclerosis; lupus nephropathy; diabetic nephropathy)	MPs levels are not changed	Daniel et al., 2006
Hemodyalisis	Elevated MPs	Daniel et al., 2006
Nephrotic syndrome	Lactahedrin^+^ ErMPs, PMPs and EMPs elevated	Gao et al., 2012
Uremia with or w/o dialysis	Elevated MPs, EMPs levels	Nomura et al., 1993; Merino et al., 2010
**TRANSPLANTATION**		
GVHD disease (allogeneic hematopoietic stem cell transplantation)	Elevated MPs, PMPs levels;	Pihusch et al., 2002; Nomura et al., 2005; 2008; Trummer et al., 2011; Rank et al., 2011; De Rop et al., 2011; Wu et al., 2012
	Elevated PSGL-1 MPs levels	
	Elevated Er-MPs levels	
	Elevated EMPs levels; decreased EMPs in early phase after allo-HSCT	
Kidney transplantation	Procoagulant MPs decreased	Al-Massarani et al., 2009
Liver transplantation	Elevated MPs levels	Brodsky et al., 2008
**OTHER**		
Acute liver injury	Elevated CD39^+^ MPs levels	Schmelzle et al., 2012
Acute respiratory distress syndrome	Elevated Leu and NeuMPs levels	Guervilly et al., 2011
Alzheimer’s disease	Elevated EMPs	Xue et al., 2012
Atopic dermatitis	Elevated PMPs levels	Tamagawa-Mineoka et al., 2009
Cystic fibrosis	Elevated levels of granulocyte MPs in sputum (CD11a^+^ and CD66b^+^)	Porro et al., 2010
Fabry disease	Elevated CD63^+^ MPs	Gelderman et al., 2007; Vedder et al., 2009
Metabolic syndrome	Different patterns of MPs levels :	Arteaga et al., 2006; Chironi et al., 2006; Agouni et al., 2008; Ueba et al., 2008; Helal et al., 2010
	Elevated EMPs, PMPs, leukocyte-derived MPs and Er-MPs levels	
Obstructive sleep apnea syndrome	PMPs elevated	Maruyama et al., 2012
Polymyalgia rheumatica	CD31^+^/CD42^-^ EMPs elevated	Pirro et al., 2011
Schizophrenia	MPs elevated in cerebrospinal liquid	Mobarrez et al., 2013

*references for Table 4 (Additional file 7).

The association of elevated levels of certain MP subtypes with specific disease states may also have therapeutic implications. An interesting possibility is the use of *in vitro* generated MPs to stimulate neovascularization in the diseases with impaired angiogenesis [179], while a different subset of MPs could be used to inhibit tumor-induced angiogenesis and, possibly, even tumor development [180]. Therapeutic strategies to reduce severity of disease may also decrease the level of circulating MPs. Thus, the level of platelet-derived MPs in diabetic patients is decreased after treatment with antioxidants such as vitamin C [181] or miglitol [182]. La Vignera et al. [183] showed that endothelial-derived MP (EMPs) level is significantly decreased in patients with erectile dysfunction after treatment with tadalafil. The concentration of erythrocyte-derived MPs (ErMPs) in patient blood correlates with severity of malaria disease and starts to decrease 24 hours after the beginning of antimalarial treatment, reaching baseline values after two weeks of treatment in patients infected with *P.vivax* and *P.malariae*, but after more prolonged therapy in patients with *P.falciparum*[184].

These findings have ignited interest to MPs as possible biomarkers for diagnostics and evaluation of efficiency of a therapeutic strategy.

### MPs in cancer

Cancer cell-derived MPs have been studied intensively in recent years, and their potential as diagnostic and prognostic tools has been described [185,186]. Tumor-derived MPs carry specific molecular markers typical for the cells of their origin, including epithelial cell adhesion molecule (EpCam), human epidermal growth receptor 2 (HER-2), CCR6, extracellular metalloproteinases (MMPs), vascular endothelial growth factor (VEGF), and some others [118,187-191]. However, many types of cancer, such as ovarian and pancreas malignancies, exhibit no specific biomarker that makes their screening or early detection difficult. Several groups have described the transfer of oncogenic proteins and chemokines between cells by tumor-derived MPs, which leads to the horizontal spread of aggressive phenotypes among tumor cells had not expressing these proteins by themselves [90,192]. MPs from cancer cells contain a variety of cell-surface receptors, cytoskeletal components and intracellular signaling proteins [192] and the concentration of tumor-derived MPs increases during tumor progression [186,189]. Peripheral blood from cancer patients contains not only cancer cell-derived MPs but also high levels of procoagulant and platelet-derived MPs [190], which may contribute to the development of clinically relevant haemostatic abnormalities in cancer patients that is referred to as Trousseau’s syndrome [193]. Reprogramming of target cells by MPs was first described by Ratajczak et al. [122], and later on it has been shown directly that exposure of normal cells to cancer cell-derived MPs that contain fibronectin and tissue transglutaminase causes the recipient cells to acquire a transformed phenotype [194]. Moreover, it was reported that when MPs produced by cultures of different human primary tumors or established tumor cell lines were isolated and added back to the same cancer cells the growth of these cells was accelerated [90]. Finally, it was found that MPs derived from a subset of CD105^+^ tumor-initiating human renal carcinoma cells were able to activate endothelial cells *in vitro* and triggered their growth and vascularization after implantation into SCID mice [195].

MPs shed by tumor cells serve as a profound additional pathway for drug release [196]. Intensity of MP shedding and anti-cancer drug resistance by positively correlate across wide number of cell lines and drugs tested [196]. Besides, Jaiswal et al. [197] have shown that MPs derived from both *ABCB1*-mediated multidrug-resistant acute lymphoblastic leukemic and breast cancer cells can transfer mRNAs that encode multidrug resistance (MDR) transporter proteins into the drug-sensitive cancer cells, allowing for horizontal acquisition of drug resistance. This study also demonstrated that MPs express greater concentration of specific miRNAs as compared to their cells of origin (for example *miR-451*). This “non-genetic” intercellular transfer of molecular components provides an alternative pathway for circumvention of MDR. The time-dependence of P-gp transfer by MPs and increase of influx activity in MCF-7 breast cancer cells reveal the occurence of multiple routes for extragenetic MDR acquisition by cancer cells [198].

The contribution of platelet-derived MPs to hematogeneous cancer metastasis is tied to their procoagulant activity [199]. Metastatic processes depend on the haemostatic competence of tumour cells and their capacity to initiate microvascular thrombosis [190], and MPs may promote these processes via transfer of mRNAs that encode angiogenic factors such as MMP-9, interleukin-8, VEGF [200]. Indeed, injection PMP-covered Lewis lung carcinoma cells (LLC) into syngeneic mice results in the formation of significantly more metastatic foci in the lungs of these animals as compared to mice injected only with LLC [200]. Also in prostate cancer patients elevated plasma PMP levels correlate with aggressiveness of tumors and poor clinical outcome [201].

### MPs and vascular diseases

Platelet-derived MPs have been extensively investigated for their ability to induce coagulation and participate in thrombosis because they display PS and other negatively charged phospholipids that provide binding sites for activated coagulation factors [202]. PMPs have significantly higher (50-100x) procoagulant activity compared even to activated platelets [87]. PMPs may regulate additional vascular pathways, including activation of endothelial cells and leukocytes, stimulation of angiogenesis, and induction of apoptosis in endothelial cells [203]. MPs released by normal endothelial cells are implicated in angiogenesis, as well as bone regeneration and mineralization *in vivo*[204-206]. MPs originating from human atherosclerotic plaques carry mature form of tumor necrosis factor (TNF)-converting enzyme metalloprotease TACE/ADAM 17, which cleaves TNF and its receptors TNF-R1 and TNF-R2 [207]. These MPs enhance shedding of TNF from cultured human cells that overexpress TNF, as well as TNFR1 shedding from HUVEC cell lines, suggesting that TACE^+^ MPs regulate the inflammatory balance in culprit atherosclerotic plaque lesion [207]. Several forms of hemolytic anemia are associated with elevated levels of MPs in plasma and concomitantly with high tissue factor (TF) activity [97,208-210]. Monocyte-derived MP levels are elevated in the plasma of paroxysmal nocturnal hemoglobinuria patients, as monocytes in these indviduals are fragile due to a deficiency in surface expression of CD55 and CD59 [209].

Since endothelial MPs from patients with metabolic disorders induce endothelial dysfunction in animal models [211], and elevated circulating MP levels are associated with both severity and adverse outcomes in several cardiovascular pathologies, including myocardial infarction, atherothrombosis, hypertension, and preeclampsia, risk stratification for these conditions now relies, in part, on the measurement of MP levels (summarized in Additional file 3).

### MPs and infectious diseases

Bacterial virulence factors such as the M1 protein from *S.pyogenes* and lipopolysaccharide (LPS) from *E.coli* stimulate the release of procoagulant MPs from PBMCs [212,213]. A number of publications have reported that specific MP subtypes in septic patients, such as endothelium-, platelet- and monocyte-derived MPs, are associated with different etiologies of sepsis (*S.pyogenes, Staphylococcus, Pneumococcus, Enterococcus*) [213,214]. Elevated MP levels are associated with systemic inflammatory response syndrome (SIRS) and hemolytic uremic syndrome caused by *E.coli* infection [215,216]. It is possible that MPs produced by infected cells, or by cells exposed to bacterial virulence factors, may contribute to secondary organ dysfunction observed during these disorders. Mastronardi and colleagues [217] have reported that injection of MPs from septic shock patients into experimental animals leads to changes in the enzyme systems related to inflammation, nitrative and oxidative stress. These findings are in accordance with the results obtained by other investigators [218], which have indicated that the injection of normal rats with MPs obtained from septic rats induces hemodynamic changes and septic inflammatory responses in the heart.

ErMP levels are significantly increased in the blood of malaria patients with coma or severe malaria [184] and correlate with plasma TNF concentrations [219]. Cell-derived and *Plasmodium*-derived MPs contribute to the development of fatal cerebral malaria [220-222]. In *in vitro* experiments PMPs were found to bind preferentially to *Plasmodium*-infected erythrocytes or iRBCs, and increase cytoadherence of iRBCs to HUVECs [222]. Moreover, it has been shown that *P.falciparum* synthesizes and packages Maurer’s clefts* (*parasite-derived structures within the host cell cytoplasm that are thought to function as a sorting compartment between the parasite and the parasitophorous membrane [223]) subsequently exporting them to the cytoplasm of infected erythrocytes via MPs shedding [223]. Observations on another eukaryotic parasite, *L.donovani*, also demonstrated that parasite-produced microvesicles are released from infected cells [224]. MPs released by bacteria *Porphyromonas gingivalis* that cause periodontitis disease, carry lipoproteins and other proinflammatory mediators to the distant sites and contribute to progression of atherosclerosis [225,226]. Summarizing it could be concluded that in many cases MPs and exosomes released by infected host cells contain pathogen-derived antigens and virulence factors and may modulate disease progression and immune response [225-230].

## Conclusion

**Figure 5:**
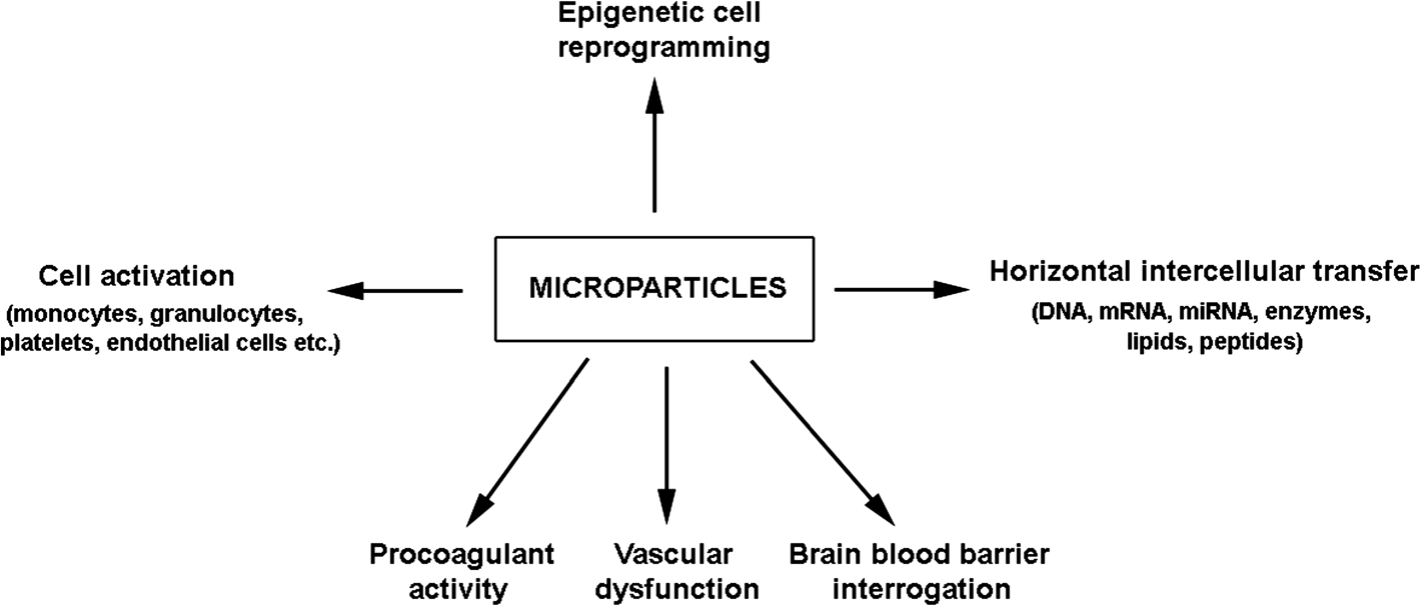
Potential mechanisms of MP action.

As methods for isolating and characterizating MPs advance, it is anticipated better understanding of the mechanisms of MP formation and functional activity will be achieved in near future (a current overview of MP activity is summarized in Figure 5). Flow cytometry, fluorescent microscopy and light scattering methods will be critical for the characterization of MP preparations. A growing number of reports have demonstrated that MPs are produced by a remarkably diverse array of cell types and may alter the phenotype and behavior of different cell populations. However, despite four decades of MP research, we are just beginning to understand the contribution of MPs to disease development and pathogenesis. The association of elevated MP levels with many different pathological states makes them of particular interest for clinical research, and suggests that these tiny vesicles have great potential for the development of new diagnostic assays aimed at identifying early stages of pathological disorders and response for therapy, the creation of a novel class of therapeutics for improved intervention in a group of difficult-to-treat diseases. Future diagnostic exploitation of MPs may circumvent the need for some current invasive procedures, such as amnioscentesis or chorion villus sampling for the diagnosis of prenatal disorders. Further dissection of circulating MP components and their functional roles will undoubtly expand their usefulness as biomarkers and, in turn, as sentinels that steer investigators to more efficacious treatment options.

## Abbreviations

AB: Antibody; ABCA1: ATP binding cassete transporter A1; ABCB1: ATP binding cassete transporter B1; ADAM10: A disintegrin and metalloproteinase domain-containing protein 10; ARF6: ADP-ribosylation factor 6; Allo-HSCT: Allogeneic hematopoietic stem cell transplantation; ARFCES: Carcinoembryonic antigen; ASCT: Allogeneic stem cell transplantation; BALF: Bronchoalveolar lavage fluid; calcein AM: Acetometoxy derivate of calcein; CCR5: C-C chemokine receptor type 5; CXCL12: Chemokine (C-X-C motif) ligand 12; CXCR-4: C-X-C chemokine receptor type 4; DC: Dendritic cell; EDTA: Ethylenediaminetetraacetic acid; EM: Electronic microscopy; EGFR: Epidermal growth factor receptor; EMPs: Endothelial microparticles; EpCAM: Epithelial cell adhesion molecule; Er-MPs: Erythrocyte-derived microparticles; ERK: Extracellular signal-regulated kinase; Fas: CD95; FasL: Fas ligand; FMD: Flow-mediated vasodilatation; GVHD: Graft-versus-host disease; HER2: Human epidermal growth receptor 2; HIV: Human immunodeficiency virus; HSP: Heat shock protein; HUVEC: Human umbilical vein endothelial cell; ICAM-1: Intercellular adhesion molecule 1; LLC: Lewis lung carcinoma; LPS: Lipopolysaccharide; MDR: Multiple drug resistance; mRNA: Messenger RNA; miRNA: microRNA; MMPs: Metalloproteinase; MPs: Microparticles; NTA: Nanoparticle tracking assay; PAF: Platelet-activating factor; PBMCs: Peripheral blood mononuclear cells; PCOS: Polycystic ovary syndrome; P-gp: P-glycoprotein; PMPs: Platelet-derived microparticles; PMNs: Polympophnonuclear neutrophils; PS: Phosphatidylserine; RNA: Ribonucleic acid; sPLA2: Secretory phospholipase A2; PTEN: Phosphatase and tensin homolog; ROCK-1: Kinase; Rho-1: Associated kinase; SIRS: Systemic inflammatory response syndrome; SLE: Systemic lupus erythematosus; STAT: Signal transducer and activator of transcription; TF: Tissue factor; TLR: Toll-like receptor; TNF-α: Tumor-necrotic factor alpha; TRAIL: TNF-related apoptosis-induced ligand; TRM: Transplantation-related mortality; TSG101: Tumor specific antigen 101; VEGF: Vascular endothelial growth factor.

## Competing interests

LD is employed by Becton Dickinson Biosciences Inc. Other authors do not have any competing interests.

## Authors’ contributions

NSB and IAV wrote the first draft. EFK, MB, JNHS, EDP and LD critically reviewed a manuscript and contributed towards figures. All authors read and approved the final manuscript.

## Supplementary Material

Additional file 1 Range of MP sizes in different publications. Click here for file

Additional file 2 **References for Table** 1 **(Summary of some methods applied for MPs research).** Click here for file

Additional file 3 MP-based risk stratification of some pathological states. Click here for file

Additional file 4 Supplemental Table. Changes in MP levels in peripheral blood of patients in response to treatments. Click here for file

Additional file 5 **References for Table** 2 **(MP levels in the plasma of healthy controls).** Click here for file

Additional file 6 **References for Table** 3 **(MP levels in the plasma and body fluids of patients with cancer).** Click here for file

Additional file 7 **References for Table** 4 **(MP levels in the plasma and body fluids of patients with different disorders).** Click here for file
