# Supplementary material for: Circulating microparticles: square the circle
Source: BMC Cell Biol. 2013 Apr 22;14:23. doi: 10.1186/1471-2121-14-23 (PMC3651414; doi:10.1186/1471-2121-14-23)
Supplement: Additional file 1 — Range of MP sizes in different publications. [file 1471-2121-14-23-S1.doc]

**Supplemental file 1. Range of MP sizes in different publications.**

| **MPs size range** | **Method of characterization** | **Reference** |
| --- | --- | --- |
| 0.5-1.0 μ | Experimental paper | Press et al, 2012 |
| 0.1-0.5 μ | Experimental paper (electronic microscopy) | Porro et al, 2010 |
| 0.1-1.0 μ | Review | Mause, Weber, 2010 |
| 0.05-1 μ | Review | Meziani et al, 2010; Martinez et al, 2011 |
| 0.15-1 μ | Review | Montoro-Garsia et al, 2011 |
| 0.1-2 μ | Review | Puddu et al, 2010 |
| < or =1.5 μ | Review | Shet, 2008 |
| 0.05-1.5 μ | Review | Roos et al, 2010 |
| 0.05-1 μ | Experimental paper | Bernimoulin et al, 2009 |
| <1 μ | Review | Siljander, 2011 |
| 0.03-1.0 μ | Review | Anderson et al, 2010 |
| 0.3-1.0 μ | Experimental paper | Prakash et al, 2012 |

**References:**

Anderson HC, Mulhall D, Garimella R: **Role of extracellular membrane vesicles in the pathogenesis of various diseases, including cancer, renal diseases, atherosclerosis, and arthritis.** *Lab Invest* 2010, **90**: 1549-1557.

Bernimoulin M, Waters EK, Foy M, Steele BM, Sullivan M, Falet H, Walsh MT, Barteneva N, Geng JG, Hartwig JH, Maguire PB, Wagner DD: **Differential stimulation of monocytic cells results in distinct populations of microparticles.** *J Thromb Haemost* 2009, **7:** 1019-1028.

Martinez MC, Tual-Chalot S, Leonetti D, Andriantsitohaina R: **Microparticles: targets and tools in cardiovascular disease.** *Trends Pharmacol Sci* 2011, **32**: 659-665.

Mause SF, Weber C: **Microparticles: protagonists of a novel communication network for intracellular information exchange.** *Circ Res* 2010, **107**: 1047-1057.

Meziani F, Delabranche X, Asfar P, Toti F: **Bench-to-bedside review: circulating microparticles-a new player in sepsis?** *Critical Care* 2010, **14**: 236.

Montoro-Garcia S, Shantsila E, Marin F, Blann A, Lip GY: **Circulating microparticles: new insights into the biochemical basis of microparticle release and activity**. *Basic Res Cardiol* 2011, **106**: 911-923.

Porro C, Lepore S, Trotta T, Castellani S, Ratclif L, Battaglino A, Di Gioia S, Martinez MC, Conese M, Maffione AB: **Isolation and characterization of microparticles in sputum from cystic fibrosis patients.** *Respir Res* 2010, **11**: 94.

Prakash PS, Caldwell CC, Lentsch AB, Pritts TA, Robinson BR: **Human microparticles generated during sepsis in patients with critical illness are neutrophil-derived and modulate the immune response**. *J Trauma Acute Care Surg* 2012, **73**: 401-406.

Press JZ, Reyes M, Pitteri SJ, Pennil C, Garcia R, Goff BA, Hanash SM, Swisher EM: **Microparticles from ovarian carcinomas are shed into ascites and promote cell migration.** *Int J Gynecol Cancer* 2012, **22**: 546-552.

Puddu P, Puddu GM, Cravero E, Muscari S, Muscari A: **The involvement of circulating microparticles in inflammation, coagulation and cardiovascular diseases.** *Can J Cardiol* 2010, **26**: 140-145.

Roos MA, Gennero L, Denysenko T, Reguzzi S, Cavallo G, Pescarmona GP, Ponzetto A: **Microparticles in physiological and in pathological conditions**. *Cell Biochem Funct* 2010, **28**: 539-548.

Shet AS: **Characterizing blood microparticles: technical aspects and challenges.** *Vasc Health Risk Manag* 2008, **4**:769.

Siljander PRM: **Platelet-derived microparticles-an updated perspective**. *Thromb Res* 2011, **Suppl 2**: S30-S33.
