# Supplementary material for: Circulating microparticles: square the circle
Source: BMC Cell Biol. 2013 Apr 22;14:23. doi: 10.1186/1471-2121-14-23 (PMC3651414; doi:10.1186/1471-2121-14-23)
Supplement: Additional file 2 — References for Table 1 (Summary of some methods applied for MPs research). [file 1471-2121-14-23-S2.doc]

**Supplemental File 4.** References for Table 1 (Summary of some methods applied for MPs research).

**References:**

Abid Hussein MN, Nieuwland R, Hau CM, Evers LM, Meesters EW, Sturk A: **Cell-derived microparticles contain caspase 3 in vitro and in vivo.** *J Thromb Haemost* 2005, **3**: 888-896.

Ayers L, Kohler M, Harrison P, Sargent I, Dragovic R, Schaap M, Nieuwland R, Brooks SA, Ferry B: **Measurement of circulating cell-derived microparticles by flow cytometry: sources of variability within the assay.** *Thromb Res* 2011, **127:** 370-377.

Bebawy M, Combes V, Lee E, Jaiswal R, Gong J, Bonhoure A, Grau GE: **Membrane microparticles mediate transfer of P-glycoprotein to drug sensitive cancer cells.** *Leukemia* 2009, **23**: 1643-1649.

Bernimoulin M, Waters EK, Foy M, Steele BM, Sullivan M, Falet H, Walsh MT, Barteneva N, Geng JG, Hartwig JH, Maguire PB, Wagner DD: **Differential stimulation of monocytic cells results in distinct populations of microparticles.** *J Thromb Haemost* 2009, **7:** 1019-1028.

Distler JH, Juengel A, Huber LC, Seemayer CA, Reich CF3rd, Gay RE, Michel BA, Fontana A, Gay S, Pisetsky DS, Distler O: **The induction of matrix metalloproteinase and cytokine expression in synovial fibroblasts stimulated with immune cell microparticles.** *Proc Natl Acad Sci*  2005, **102**: 2892-2897.

Duarte TA, Noronha-Dutra AA, Nery JS, Ribeiro SB, Pitanga TN, Lapa e Silva JR, Arruda S, Boechat N: ***Mycobacterium tuberculosis*-induced neutrophil ectosomes decrease macrophage activation.** *Tuberculosis(Edinb)* 2012, **92**: 218-225.

Gercel-Taylor C, Atay S, Tullis RH, Kesimer M, Taylor DD: **Nanoparticle analysis of circulating cell-derived vesicles in ovarian cancer patients.** *Anal Biochem* 2012, **428**: 44-53.

Hess C, Sadallah S, Hefti A, Landmann R, Schifferli JA: **Ectosomes released by human neutrophils are specialized functional units.** *J Immunol* 1999, **163:** 4564-4573.

Jy W, Horstmann LL, Jimenez JJ, Ahn JS, Biro E, Nieuwland R, Sturk A, Dignat-George F, Sabatier F, Camoin-Jau L, Sampol J, Hugel B, Zobairi F, Freyssinet JM, Nomura S, Shet AS, Key NS, Hebbel RP: **Measuring circulating cell-derived microparticles**. *J Thromb Haemost* 2004, **2**: 1842-1843.

Lawrie AS, Albanyan A, Cardigan RA, Mckie IJ, Harrison P: **Microparticle sizing by dynamic light scattering in fresh-frozen plasma.** *Vox Sang* 2009, **96**: 206-212.

Leong HS, Podor TJ, Manocha B, Lewis JD: **Validation of flow cytometric detection of platelet microparticles and liposomes by atomic force microscopy.** *J Thromb Haemost* 2011, **9**: 2466-2476.

Leroyer AS, Isobe H, Leseche G, Castier Y, Wassef M, Mallat Z, Binder BR, Tedgui A, Boulanger CM: **Cellular origins and thrombogenic activity of microparticles isolated from human atherosclerotic plaques.** *J Am Coll Cardiol* 2007, **49**: 772-777.

Lima LG, Chammas R, Monteiro RQ, Moreira ME, Barcinski MA: **Tumor-derived microvesicles modulate the establishment of metastatic melanoma in a phosphatidylserine-dependent manner.** *Cancer Lett* 2009, **283:** 168-175.

Manly DA, Wang J, Glover SI, Kasthuri R, Liebman HA, Key NS, Mackman N: **Increased microparticle tissue factor activity in cancer patients with venous thromboembolism.** *Thromb Res* 2009, **125**: 511-512.

Mayr M, Grainger D, Mayr U, Leroyer AS, Leseche G, Sidibe A, Herbin O, Yin X, Gomes A, Madhu B, Griffiths JR, Xu Q, Tedgui A, Boulanger CM: **Proteomics, metabolomics, and immunomics on microparticles derived from human atherosclerotic plaques.** *Circ Cardiovasc Genet* 2009, **2**: 379-388.

Nantakomol D, Palasuwan A, Chaowanathikhom M, Soogarun S, Imwong M: **Red cell and platelet-derived microparticles are increased in G6PD-deficient subjects.** *Eur J Hematol* 2012, **89**: 423-429.

Orozco AF, Lewis DE: **Flow cytometric analysis of circulating microparticles in plasma.** *Cytometry A* 2010, **77**: 502-514.

Porro C, Lepore S, Trotta T, Castellani S, Ratclif L, Battaglino A, Di Gioia S, Martinez MC, Conese M, Maffione AB: **Isolation and characterization of microparticles in sputum from cystic fibrosis patients.** *Respir Res* 2010, **11**: 94.

Rood IM, Deegens JKJ, Merchant ML, Tamboer WPM, Wilkey DW, Wetzels JFM, Klein JB: **Comparison of three methods for isolation of urinary microvesicles to identify biomarkers of nephrotic syndrome.** *Kidney Int* 2010, **78:** 810-816.

Salzer U, Zhu R, Luten M, Isobe H, Pastushenko V, Perkmann T, Hinterdorfer P, Bosman GJ: **Vesicles generated during storage of red cells are rich in the lipid raft marker stomatin.** *Transfusion* 2008, **48**: 451-462.

Sander TL, Ou JS, Densmore JC, Kaul S, Matus I, Twigger S, Halligan B, Greene AS, Pritchard KA, Oldham KT: **Protein composition of plasminogen activator type 1-derived endothelial microparticles**. *Shock* 2008, **29**: 504-511.

Shah MD, Bergeron AL, Dong JF, Lopez JA: **Flow cytometric measurement of microparticles: pitfalls and protocol modifications**. *Platelets* 2008, **19**: 365-372.

Tesselaar ME, Romijin FP, Van Der Linden IK, Prins FA, Bertina RM, Osanto S: **Microparticle-associated tissue factor activity: a link between cancer and thrombosis?** *J Thromb Haemost* 2007, **5:** 520-527.

van der Heyde HC, Gramaglia I, Combes V, George TC, Grau GE: **Flow cytometric analysis of microparticles**. *Methods Mol Biol* 2011, **699**: 337-354.

Van Der Pol E, Hoekstra AG, Sturk A, Otto C, Van Leeuwen TG, Niewland R: **Optical and non-optical methods for detection and characterization of microparticles and exosomes.** *J Thromb Haemost* 2010, **8**: 2596-2607.

Witek RP, Yang L, Liu R, Jung Y, Omenetti A, Syn W-K, Choi SS, Cheong Y, Fearing CM, Agboola KM, Chen W, Diehl AM: **Liver cell-derived microparticles activate hedgehog signaling and alter gene expression in hepatic endothelial cells.** *Gastroenterology* 2009, **136**: 320-330.

Xu Y, Nakane N, Maurer-Spurej E: **Novel test for microparticles in platelet-rich plasma and platelet concentrates using dynamic light scattering**. *Transfusion* 2010, **51**: 363-370.

Yuana Y, Oosterkamp TH, Bahatyrova S, Ashcroft B, Garcia Rodriquez P, Bertina RM, Osanto S: **Atomic force microscopy: a novel approach to the detection of nanosized blood microparticles.** *J Thromb Haemost*, 2010, **8**: 315-323.

Yuana Y, Bertina RM, Osanto S: **Pre-analytical and analytical issues in the analysis of blood microparticles.** *Thromb Haemost* 2011, **105**: 396-408.

Zwicker JI: **Impedance-based flow cytometry for the measurement of microparticles.** *Semin Thromb Hemost* 2010, **36**: 819-823.
