# Supplementary material for: Circulating microparticles: square the circle
Source: BMC Cell Biol. 2013 Apr 22;14:23. doi: 10.1186/1471-2121-14-23 (PMC3651414; doi:10.1186/1471-2121-14-23)
Supplement: Additional file 3 — MP-based risk stratification of some pathological states. [file 1471-2121-14-23-S3.doc]

**Supplemental file 2. MPs-based risk stratification of some pathological states.**

| **Pathological state** | **MPs** | **Biomarker** | **Reference** |
| --- | --- | --- | --- |
| Functional status of the transplanted liver | Circulating MPs | Increased in patients with complications | Brodsky et al, 2008 |
| Risk-stratification of patients with allogeneic stem-cell transplantation (ASCT) | TF^+^MPs | Increased in patients with complications | De Rop et al, 2011 |
| Risk-stratification of patients with allogeneic stem-cell transplantation (ASCT) | Er-MPs level | Increased in patients with graft-vs-host disease | Rank et al, 2011 |
| Early development of emphysema | EMP levels (CD42b^-^/CD31^+^ ) | Increased in patients with early lung destruction | Gordon et al, 2011 |
| Diabetes | CD36, putative diabetic plasma marker | Associated with MPs | Alkhatbeh et al, 2011 |
| Acute respiratory distress syndrome | Leu-MPs level | Increased in survivors | Guervilly et al, 2011 |
| Risk-stratification of patients with stable coronary disease | CD31^+^Annexin V^+^ | Increased in patients with cardiobascular risk factors | Werner et al, 2006; Sinning et al, 2011 |
| Risk-stratification of patients with heart failure | CD144^+^ EMP | Biomarker of endothelial disfunction | Nozaki et al, 2010 |
| Risk-stratification of patients with pulmonary hypertension | CD62e^+^ EMPs | Elevated levels are associated with poor outcome | Amabile et al, 2009 |
| Risk stratification of menopausal women for premature coronary calcification | PMPs and EMPs | Increased in early menopausal women with premature CAC | Jayachandran et al, 2008 |
| Risk-stratification of patients with spontaneous subarachnoidal hemorrhage | EMPs | Increased in patients with symptomatic cerebral vasospasm | Lackner et al, 2010 |
| Risk-stratification of patients with chronic renal failure | EMPs, CD144^+^ and CD146^+^ | Increased in patients with endothelial dysfunction in uremia | Faure et al, 2006 |
| Risk-stratification of patients with cerebrovascular disease | PECAM-1/CD31^+^ EMPs | Do not discriminate between stroke mimics and acute ischemic stroke | Williams et al, 2007 |
| Risk-stratification of patients with cerebrovascular disease | CD62^+^ EMPs | Increased risk for ischemic stroke | Jung et al, 2009 |
| Risk-stratification of patients with intracerebral hemorrhage | Plasma or cerebrospinal fluid levels of procoagulant MPs | High levels of MPs correlate with poor outcome | Huang et al, 2009 |
| Henoch-Schönlein purpura | CD144^+^ and CD146^+^ EMPs | Surrogate marker for subclinical inflammation | Dursun et al, 2010 |
| Diabetes mellitus | EMPs, CD31^+^, CD105^+^ and CD106^+^ | Elevated in patients with DM-independent predictor | Tramontano et al, 2010 |
| Novel diagnostic marker | T-cell MPs | Increased in hepatitis C and correlate with severity of disease | Kornek et al, 2011 |
| Marker for cerebral malaria | EMPs | Elevated | Pankoui Mfonkeu et al, 2010 |
| Endotoxemia (*E.coli* endotoxin) | PMPs | Associated with poor prognosis | Eriksson et al 1998 |

**References:**

Amabile N, Heiss C, Chang V, Angeli FS, Damon L, Rame EJ, McGlothlin D, Grossman W, De Marco T, Yeghiazarians Y: **Increased CD62e^+^ endothelial microparticles levels predict poor outcome in pulmonary hypertension patients.** *J Heart Lung Transplant* 2009, **28:** 1081-1086.

Alkhatbeh MJ, Mhaidat NM, Enieti AK, Lincz LF, Thorne RF: **The putative diabetic plasma marker, soluble CD36, is non-cleaved, non-soluble and entirely associated with microparticles.** *J Thromb Haemost* 2011, **9:** 844-851.

Brodsky SV, Facciuto ME, Heydt D, Chen J, Islam HK, Kaistura M, Ramaswamy G, Aguero-Rosenfeld M: **Dynamics of circulating microparticles in liver transplant patients.** *J Gastrointestin Liver Dis* 2008, **17:** 261-268.

De Rop C, Stadler M, Buchholz S, Eisert R, Ganser A, Trummer A. **Evaluation of** **tissue factor bearing microparticles as biomarkers in allogeneic stem-cell transplantation.** *Transplantation* 2011, **92:** 351-358.

Eriksson M, Nelson D, Nordgren A, Larsson A: **Increased platelet microvesicle formation is associated with mortality in a porcine model of endotoxemia.** *Acta Anaesthesiol Scand* 1998, **42**: 551-557.

Faure V, Dou L, Sabatier F, Cerini C, Sampol J, Berland Y, Brunet P, Dignat-George F: **Elevation of circulating endothelial microparticles in patients with chronic renal failure.** *J Thromb Haemost* 2006, **4:** 566-573.

Gordon C, Gudi K, Krause A, Sackrowitz R, Harvey BG, Strulovici-Barel Y, Mezey JG, Crystal RG: **Circulating endothelial microparticles as a measure of early lung destruction in cigarette smokers.** *Am J Respir Crit Care Med* 2011, **184:** 224-232.

Guervilly C, Lacroix R, Forel JM, Roch A, Camoin-Jau L, Papazian L, Dignat-George F: **High levels of circulating leukocyte microparticles are associated with better outcome in acute respiratory distress syndrome.** *Crit Care* 2011, **15:** R31.

Dursun I, Duesuensel R, Poyrazoglu HM, Gunduz Z, Patiroglu T, Ulger H, Gurgoze MK: **Circulating endothelial microparticles in children with Henoch-Schoenlein purpura; preliminary results.** *Rheumatol Int* 2011, **31**:1595-1600.

Huang M, Hu YY, Dong XQ: **High concentrations of procoagulant microparticles in the cerebrospinal fluid and peripheral blood of patients with acute basal ganglia hemorrhage are associated with poor outcome.** *Surg Neurol* 2009, **72:** 481-489.

Jayachanadran M, Litwiller RD, Owen WG, Heit JA, Behrenbeck T, Mulvagh SL, Araoz PA, Budoff MJ, Harman SM, Miller VM: **Characterization of blood borne microparticles as markers of premature calcification in newly menopausal women.** *Am J Physiol Heart Circ Physiol* 2008, **295:** H931-H938.

Jung KH, Chu K, Lee ST, Park HK, Bahn JJ, Kim DH, Kim JH, Kim M, Kun Lee S, Roh JK: **Circulating endothelial microparticles as a marker of cerebrovascular disease**. *Ann Neurol* 2009, **66**: 191-199.

Kornek M, Lynch M, Mehta SH, Lai M, Exley M, Afdahl NH, Schuppan D. **Circulating microparticles as disease-specific biomarkers of severity of inflammation in patients with hepatitis C or nonalcoholic steatohepatitis.** *Gastroenterology* 2012, 143: 448-458.

Lackner P, Dietmann A, Beer R, Fischer M, Broessner G, Helbok R, Marxgut J, Pfausler B, Schmutzhard E: **Cellular microparticles as a marker for cerebral vasospasm in spontaneous subarachnoid hemorrhage.** *Stroke* 2010, **41:** 2353-2357.

Nozaki T, Sugiyama S, Sugamura K, Ohba K, Matsuzawa Y, Konishi M, Matsubara J, Akiyama E, Sumida H, Matsui K, Jinnouchi H, Ogawa H: **Prognostic value of endothelial microparticles in patients with heart failure.** *Eur J Heart Fail* 2010, **12:** 1223-1228.

Pankoui Mfonkeu JB, Gouado I, Fotso Kuate H, Zambou O, Amvam Zoll PH, Grau GE, Combes V: **Elevated cell-specific microparticles are a biological marker for cerebral dysfunctions in human severe malaria**. *PLoS One* 2010, **5**: e13415.

Rank A, Nieuwland R, Toth B, Pihusch V, Delker R, Hiller E, Kolb HJ, Pihusch R: **Microparticles for diagnosis of graft-versus-host disease after allogeneic stem transplantation.** *Transplantation* 2011, **92:** 244-250.

Sinning JM, Losch J, Walenta K, Boehm M, Nickenig G, Werner N: **Circulating CD31^+^/Annexin V^+^ microparticles correlate with cardiovascular outcomes.** *Eur Heart J* 2011, **32:** 2034-2041.

Tramontano AF, Lyubarova R, Tsiakos J, Palaia T, Deleon JR, Ragolia L: **Circulating endothelial microparticles in diabetes mellitus.** *Mediators Inflamm* 2010, 2010:250476, doi: 10.1155/2010/25047.

Williams JB, Jauch EC, Lindsell CJ, Campos B. **Endothelial microparticle levels are similar in acute ischemic stroke and stroke mimics due to activation and not apoptosis/necrosis**. *Acad Emerg Med* 2007, **14:** 685-690.

Werner N, Wassmann S, Ahlers P, Kosiol S, Nickenig G: **Circulating CD31+/annexin V+ apoptotic microparticles correlate with coronary endothelial function in patients with coronary artery disease.** *Arterioscler Thromb Vasc Biol* 2006, **26**: 112-116.
