# Supplementary material for: Circulating microparticles: square the circle
Source: BMC Cell Biol. 2013 Apr 22;14:23. doi: 10.1186/1471-2121-14-23 (PMC3651414; doi:10.1186/1471-2121-14-23)
Supplement: Additional file 4 — Supplemental Table. Changes in MP levels in peripheral blood of patients in response to treatments. [file 1471-2121-14-23-S4.doc]

**Supplemental file 3.** Changes in MPs levels in peripheral blood of patients in response to treatments.

| **Microparticles** | **Disease/Stimul** | **Drug** | **Reference** |
| --- | --- | --- | --- |
| ErMPs decreased | Sickle disease | Hydroxyurea | Gerotziafas et al, 2012 |
| EMPs decreased | Polymyalgia rheumatica | Corticosteroids | Pirro et al, 2011 |
| MPs decreased | MPs production in response to LPS *N.meningitidis* | IL-10 | Ovstebo et al, 2011 |
| EMPs decreased | Erectile disfunction and metabolic syndrome | Tadalafil, inhibitor of phosphodiesterase V | La Vignera, 2011a |
| PMPs and myeloblast-derived MPs declined | Acute myeloid leukemia | Chemotherapy | Van Aalderen et al, 2011 |
| MPs expressing complement components, CRP or serum amyloid-P(SAP) not altered | Rheumatoid arthritis | Anti-inflammatory therapy | Van Eijk et al, 2010 |
| EMPs decreased | Diabetus mellitus | Eicosapentaenoic acid | Nomura et al, 2009 |
| PMPs increased | Post-menopausal women | Hormone-replacement therapy | Rank et al, 2012 |
| PMPs and EMPs decreased | Coronary artery disease | Acetylsalicylate | Bulut et al, 2011 |
| PMPs and EMPs (CD31^+^CD41^+^ and CD31^+^CD41^-^) decreased | Cancer | Cisplatin | Periard et al, 2007 |
| PMPs decreased | Cardiopulmonary bypass | Nitric oxide gas and iloprost | Chung et al, 2005 |
| PS^+^ MPs decreased | Crohn's disease | Infiximab | Chamouard et al, 2005 |
| PMPs and MMPs decreased | Diabetes mellitus | Ticlopidine | Shouzu et al, 2004 |

**References:**

Bulut D, Becker V, Muegge A: **Acetylsalicylate reduces endothelial and platelet-derived microparticles in patients with coronary artery disease.** *Can J Physiol Pharmacol* 2011, **89**: 239-244.

Chamouard P, Desprez D, Hugel B, Kunzelmann C, Gidon-Jeangirard C, Lessard M, Baumann R, Freyssinet JM, Grunebaum L: **Circulating cell-derived microparticles in Crohn's disease.** *Dig Dis Sci* 2005, **50:** 574-580.

Chung A, Wildhirt SM, Wang S, Koshal A, Radomski MW: **Combined administration of nitric oxide gas and iloprost during cardiopulmonary bypass reduces platelet dysfunction: a pilot clinical study.** *J Thorac Cardiovasc Surg* 2005, **129**: 782-790.

Gerotziafas GT, Van Dreden P, Chaari M, Galea V, Khaterchi A, Lionnet F, Stankovic-Stojanovic K, Blanc-Brude O, Woodhams B, Maier-Redelsperger M, Girot R, Hatmi M, Elalamy I: **The acceleration of the propagation phase of thrombin generation in patients with steady-state sickle cell disease is associated with circulating erythrocyte-derived microparticles.** *Thromb Haemost* 2012, **107**: 1044-1052.

La Vignera S: **New immunophenotype of circulating endothelial progenitor cells and endothelial microparticles in patients with erectile dysfunction and metabolic syndrome: effects of taladafil administration.** *Int Angiol* 2011, **30:** 415-423.

Nomura S, Shouzu A, Omoto S, Inami N, Ueba T, Urase F, Maeda Y: **Effects of eicosapentaenoic acid on endothelial cell–derived microparticles, angiopoietins and adiponectin in patients with type 2 diabetes**. *J Atheroscler Thromb* 2009, **16**: 83-90.

Ovstebo R, Aass HC, Haug KB, Troseid AM, Gopinathan U, Kierulf P, Berg JP, Brandtzaeg P, Henriksson CE: **LPS from *Neisseria meningitidis* is crucial for inducing monocyte- and microparticle-associated tissue factor activity but not for tissue factor expression.** *Innate Immun* 2012, 18:580-591.

Periard D, Boulanger CM, Eyer S, Amabile N, Pugin P, Gerscheimer C, Hayoz D: **Are circulating endothelial-derived and platelet-derived microparticles a pathogenic factor in the cisplatin-induced stroke?** *Stroke* 2007, **38**: 1636-1638.

Pirro M, Bocci EB, Di Filippo F, Schillaci G, Mannarino MR, Bagaglia F, Gerli R, Mannarino E: **Imbalance between endothelial injury and repair in patients with polymyalgia rheumatica: improvement with corticosteroid treatment**. *J Intern Med* 2012, **272**: 177-184.

Rank A, Nieuwland R, Nikolajek K, Roesner S, Wallwiener LM, Hiller E, Toth B: **Hormone replacement therapy leads to increased plasma levels of platelet derived microparticles in postmenopausal women.** *Arch Gynecol Obstet* 2012, **285**: 1035-1041.

Shouzu A, Nomura S, Omoto S, Hayakawa T, Nishikawa M, Iwasaka T: **Effect of ticlopidine on monocyte-derived microparticles and activated platelet markers in diabetes mellitus.** *Clin Appl Thromb Hemost* 2004, **10**: 167-173.

Van Aalderen MC, Trappenburg MC, Van Schilfgaarde M, Molenaar PJ, Ten Cate H, Terpstra WE, Leyte A: **Procoagulant myeloblast-derived microparticles in AML-patients: changes in numbers and thrombin generation potential during chemotherapy.** *J Thromb Haemost* 2011, **9:** 223-226.

Van Eijk IC, Tushuizen ME, Sturk A, Dijkmans BA, Boers M, Voskuyl AE, Diamant M, Wolbink GJ, Nieuwland R, NurmohAMED mt: **Circulating microparticles remain associated with complement activation despite intensive anti-inflammatory therapy in early rheumatoid arthritis**. *Ann Rheum Dis* 2010, **69**: 1378-1382.
