# Supplementary material for: Circulating microparticles: square the circle
Source: BMC Cell Biol. 2013 Apr 22;14:23. doi: 10.1186/1471-2121-14-23 (PMC3651414; doi:10.1186/1471-2121-14-23)
Supplement: Additional file 5 — References for Table 2 (MP levels in the plasma of healthy controls). [file 1471-2121-14-23-S5.doc]

**Supplemental file 5.** References for Table 2 (MPs levels in the plasma of healthy controls).

Aras O, Shet A, Bach RR, Hysjulien JL, Slungaard A, Hebbel RP, Escolar G, Jilma B, Key NS: **Induction of microparticle- and cell-associated intravascular tissue factor in human endotoxemia.** *Blood* 2004, **103:** 4545-4553.

Berckmans RJ, Neiuwland R, Boing AN, Romijn FP, Hack CE, Stark A. **Cell-derived microparticles circulate in healthy humans and support low grade thrombin generation.** *Thromb Haemost* 2001, **85**: 639-646.

Bretelle F, Sabatier F, Desprez D, Camoin L, Grunebaum L, Combes V, D'Ercole C, Dignat-George F. **Circulating microparticles: a marker of procoagulant state in normal pregnancy and pregnancy complicated by preeclampsia or intrauterine growth restriction.** *Thromb Haemost* 2003*,* **89**: 486-492.

Caby MP, Lankar D, Vincendeau-Scherrer C, Raposo G, Bonnerot C: **Exosomal-like vesicles are present in human blood plasma.** *Int Immunol* 2005, **17**:879-887.

Chaar V, Romana M, Tripette J, Broguere C, Huisse MG, Hue O, Hardy-Dessources MD, Connes P: **Effect of strenuous physical exercise on circulating cell-derived microparticles.** *Clin Hemorheol Microcirc* 2011, **47:** 15-25.

Esposito K, Ciotala M, Schisano B, Gualdiero R, Sardelli L, Misso L, Giannetti G, Giuqliano D: **Endothelial microparticles correlate with endothelial dysfunction in obese women.** *J Clin Endocrinol Metab* 2006, **91:** 3676-3679.

Forest A, Pautas E, Ray P, Bonnet D, Verny M, Amabile N, Boulanger C, Riou B, Tedgui A, Mallat Z, Boddaert J: **Circulating microparticles and procoagulant activity in elderly patients.** *J Gerontol A Biol Sci Med Sci* 2010, **65**: 414-420.

Goichot B, Grunebaum L, Desprez D, Vinzio S, Meyer L, Schlienger JL, Lessard M, Simon C: **Circulating procoagulant microparticles in obesity.** *Diab Metab* 2006, **32:** 82-85.

Gordon C, Gudi K, Krause A, Sackrowitz R, Harvey BG, Strulovici-Barel Y, Mezey JG, Crystal RG: **Circulating endothelial microparticles as a measure of early lung destruction in cigarette smokers.** *Am J Respir Crit Care Med* 2011, **184:** 224-232.

Grant R, Ansa-Addo E, Stratton D, Antwi-Baffour S, Jorfi S, Kholia S, Krige L, Lange S, Inal J: **A filtration-based protocol to isolate human plasma membrane-derived vesicles and exosomes from blood plasma.** *J Immunol Methods* 2011, **371**: 143-151.

Gunduz Z, Dursun I, Tulpar S, Bastug F, Baykan A, Yikilmaz A, Patiroglu T, Poyrazoglu HM, Akin L, Yel S, Dusunsel R: **Increased endothelial microparticles in obese and overweight children**. *J Pediatr Endocrinol Metab* 2012, **25**: 1111-1117.

Proulle V, Hugel B, Guillet B, Grunebaum L, Lambert T, Freyssinet JM, Dreyfus M: **Circulating microparticles are elevated in haemophiliacs and non-haemophilic individuals aged <18 years.** *Br J Haematol* 2005, **131:** 487-489.

Rank A, Nieuwland R, Roesner S, Nikolajek K, Hiller E, Toth B: **Climacteric lowers plasma levels of platelet-derived microparticles: a pilot study in pre-versus postmenopausal women.** *Acta Haematol* 2012, **128**: 53-59.

Schweintzger S, Schlagenhauf A, Rinner B, Bernhard H, Novak M, Leschnik B, Muntean W: **High microparticle concentration in cord plasma.** *Hamostaseologie* 2010, **Suppl 1:**S122-S125.

Schweintzger S, Schlagenhauf A, Leschnik B, Rinner B, Bernhard H, Novak M, Muntean W: **Microparticles in newborn cord blood: slight elevation after normal delivery.** *Thromb Res* 2011, **128:** 62-67.

Strohacker K, Breslin WL, Carpenter KC, Davidson TR, Agha NH, McFarlin BK: **Moderate-intensity, pre-meal cycling blunts postprandial increases in monocyte cell surface CD18 and CD11a and endothelial microparticles following a high-fat meal in young adults.** *Appl Physiol Nutr Metab* 2012, **37**: 530-539.

Toth B, Nikolajek K, Rank A, Nieuwland R, Lohse P, Pihusch V, Friese K, Thaler CJ: **Gender-specific and menstrual cycle dependent differences in circulating microparticles.** *Platelets* 2007, **18:** 515-521.

Uszynski M, Zekanowska E, Uszynski W, Kuczynski J, Zylinski A: **Microparticles (MPs), tissue factor (TF) and tissue factor inhibitor (TFPI) in cord blood plasma. A preliminary study and literature survey of procoagulant properties of MPs**. *Eur J Obstet Gynecol Reprod Biol* 2011, **158**: 37-41.

Woei-A-Jin FJSH, de Kruif MD, Rodriguez PG, Osanto S, Bertina RM: **Microparticles expressing tissue factor are concurrently released with markers of inflammation and coagulation during human endotoxemia.** *J Thromb Hemost* 2012, 10: 1185-1188.
