# Supplementary material for: Circulating microparticles: square the circle
Source: BMC Cell Biol. 2013 Apr 22;14:23. doi: 10.1186/1471-2121-14-23 (PMC3651414; doi:10.1186/1471-2121-14-23)
Supplement: Additional file 6 — References for Table 3 (MP levels in the plasma and body fluids of patients with cancer). [file 1471-2121-14-23-S6.doc]

**Supplemental file 6.** References for Table 3 (MPs levels in the plasma and body fluids of patients with cancer).

Auwerda JJ, Yuana Y, Osanto S, de Maat MP, Sonneveld P, Bertina RM, Leebek FW: **Microparticle-associated tissue factor activity and venous thrombosis in multiple myeloma.** *Thromb Haemost* 2011, **105:** 14-20.

Baran J, Baj-Krzyworzeka M, Weglarczuk K, Szatanek R, Zembala M, Barbasz J, Czupryna A, Szczepanik A, Zembala M: **Circulating tumour-derived microvesicles in plasma of gastric cancer patients.** *Cancer Immunol Immunother* 2010, **59**: 841-850.

Brodsky SV, Faccuito ME, Heydt D, Chen J, Islam HK, Kajstura M, Ramaswamy G, Aguero-Rosenfeld M: **Dynamics of circulating microparticles in liver transplant patients.** *J Gastrointestin Liver Dis* 2008, **17:** 261-268.

Coumans FA, Doggen CJ, Attard G, de Bono JS, Terstappen LW: **All circulating EpCAM^+^CK^+^CD45^-^ objects predict overall survival in castration-resistant prostate cancer.** *Ann Oncol* 2010, **21**: 1851-1857.

Fleitas T, Martinez-Sales V, Vila V, Reganon E, Mesado D, Martin M, Gomez-Codina J, Montalar J, Reynes G: **Circulating endothelial cells and microparticles as prognostic markers in advanced non-small cell lung cancer.** *PLoS One* 2012, **7**: e47365.

Ginestra A, Miceli D, Dolo V, Romano FM, Vittorelli ML: **Membrane vesicles in ovarian cancer fluids: a new potential marker.** *Anticancer Res* 1999, **19:** 3439-3445.

Ghosh AK, Secreto CR, Knox TR, Ding W, Mukhopadhyay D, Kay NE: **Circulating microparticles in B-cell chronic lymphocytic leukemia can stimulate marrow stromal cells: implications for disease progression.** *Blood* 2010, **115:** 1755-1764.

Haubold K, Rink M, Spath B, Friedrich M, Chun FK, Marx G, Amirkhosravi A, Francis JL, Bokemeyer C, Eifrig B, Langer F: **Tissue factor procoagulant activity of plasma microparticles in patients with early-stage prostate cancer.** *Thromb Haemost* 2009, **101**: 1147-1155.

Hron G, Kollars M, Weber H, Sagaster V, Quehenberger P, Eichinger S, Kyrle PA, Weltermann A:**Tissue-factor-positive microparticles: cellular origin and association with coagulation activation in patients with colorectal cancer.** *Thromb Haemost* 2007, **97**: 1119-123

Kalinkovich A, Tavor S, Avigdor A, Kahn J, Brill A, Petit I, Goichberg P, Tesio M, Netzer N, Naparstek E, Hardan I, Nagler A, Resnick I, Tsimanis A, Lapidot T: **Functional CXCR4-expressing microparticles and SDF-1 correlate with circulating acute myelogenous leukemia cells**. *Cancer Res* 2006, **66**: 11013-11020.

Kanazawa S, Nomura S, Kuwana M, Muramutsu M, Yamaguchi K, Fukuhara S: **Monocyte-derived microparticles may be a sign of vascular complication in patients with lung cancer.** *Lung Cancer* 2003, **39:** 145-149.

Kim HK, Song KS, Park YS, Kang YH, Lee YJ, Lee KR, Kim HK, Ryu KW, Bae JM, Kim S: **Elevated levels of circulating platelet microparticles, VEGF, IL-6 and RANTES in patients with gastric cancer: possible role of a metastasis predictor.** *Eur J Cancer* 2003, **39:** 184-191.

Liebhardt S, Ditsch N, Nieuwland R, Rank A, Jeschke U, Von Koch F, Friese K, Toth B: **CEA-, Her2/neu-, BCRP- and Hsp27-positive microparticles in breast cancer patients.** *Anticancer Res* 2010, **30:** 1707-1712.

Lima LG, Chammas R, Monteiro RQ, Moreira ME, Barcinski MA: **Tumor-derived microvesicles modulate the establishment of metastatic melanoma in a phosphatidylserine-dependent manner.** *Cancer Lett* 2009, **283:** 168-175.

Ma G, Liu F, Lv L, Gao Y, Su Y: **Increased promyelocytic-derived microparticles: a novel potential factor for coagulopathy in acute promyelocytic leukemia.** *Ann Hematol* 2013, Epub Jan 24.

Manly DA, Wang J, Glover SL, Kasthuri R, Liebman HA, Key NS, Mackman N: **Increased microparticle tissue factor activity in cancer patients with venous thromboembolism.** *Thromb Res* 2010, **125:** 511-512.

Press JZ, Reyes M, Pitteri SJ, Pennil C, Garcia R, Goff BA, Hanash SM, Swisher EM: **Microparticles from ovarian carcinomas are shed into ascites and promote cell migration.** *Int J Gynecol Cancer* 2012, **22**: 546-552.

Rank A, Liebhardt S, Zwirner J, Burges A, Nieuwland R, Toth B. **Circulating microparticles in patients with benign and malignant ovarian tumors**. *Anticancer Res* 2012, **32**: 2009-2014.

Sartori MT, Della Puppa A, Ballin A, Saggiorato G, Bernardi D, Padoan A, Scienza R, d'Avella D, Cella G: **Prothrombotic state in glioblastoma multiforme: an evaluation of the procoagulant activity of circulating microparticles*.*** *J Neurooncol* 2011, **104**: 225-231

Savasan S, Buyukavci M, Buck S, Ravindranath Y: **Leukaemia/ lymphoma cell microparticles in mature B-cell neoplasms.** *J Clin Pathol* 2004, **57:** 651-653.

Smalley DM, Sheman NE, Nelson K, Theodorescu D: **Isolation and identification of potential urinary microparticle biomarkers of bladder cancer.** *J Proteome Res* 2008, **7:** 2088-2096.

Szczepanski MJ, Szajnik M, Welsh A, Whiteside TL, Boyiadzis M: **Blast-derived microvesicles in sera from patients with acute myeloid leukemia suppress natural killer cell function via membrane-associated transforming growth factor-beta1.** *Hematologica* 2011, **96:**1302-1309.

Taylor DD, Lyons KS, Gercel-Taylor C: **Shed membrane fragment-associated markers for endometrial and ovarian cancers.** *Gynecol Oncol* 2002, **84:** 443-448.

Taylor DD, Gercel-Taylor C. **MicroRNA signatures of tumor-derived exosomes as diagnostic biomarkers of ovarian cancer.** *Gynecol Oncol* 2008, **110:**13-21.

Tesselaar ME, Romijin FP, Van Der Linden IK, Prins FA, Bertina RM, Osanto S: **Microparticle-associated tissue factor activity: a link between cancer and thrombosis?** *J Thromb Haemost* 2007, **5:** 520-527.

Thaler J, Ay C, Weinstabl H, Dunkler D, Simanek R, Vormittag R, Freyssinet JM, Zielinski C, Pabinger I: **Circulating procoagulant microparticles in cancer patients.** *Annal Hematol* 2011, **90**: 447-453.

Thaler J, Ay C, Mackman N, Bertina RM, Kaider A, Marosi C, Key NS, Barcel DA, Scheithauer W, Kornek G, Zielinski C, Pabinger A: **Microparticle-associated tissue factor activity, venous thromboembolism and mortality in pancreatic, gastric, colorectal and brain cancer patients.** *J Thromb Haemost* 2012, **10**: 1363-1370.

Toth B, Liebhardt S, Steinig K, Ditsch N, Rank A, Bauerfeind I, Spannagl M, Friese K, Reininger AJ. **Platelet-derived microparticles and coagulation activation in breast cancer patients**. *Thromb Haemost* 2008, **100**: 663-669.

Trappenburg MC, van Schilfgaarde M, Bredewold EO, van Aalderen MC, Spronk HM, Ten Cate H, Leyte A, Terpstra WE: **Elevated numbers and altered subsets of procoagulant microparticles in breast cancer patients using endocrine therapy.** *Thromb Res* 2011, **127:**363-369.

Van Aalderen MC, Trappenburg MC, Van Schilfgaarde M, Molenaar PJ, Ten Cate H, Terpstra WE, Leyte A: **Procoagulant myeloblast-derived microparticles in AML-patients: changes in numbers and thrombin generation potential during chemotherapy.** *J Thromb Haemost* 2011, **9:** 223-226.

Zahra S, Anderson JA, Stirling D, Ludlam CA: **Plasma microparticles are not elevated in fresh plasma from patients with gynaecological malignancy- an observational study.** *Gynecol Oncol* 2011, **123**: 152-156.

Zwicker JI, Liebman HA, Neuberg D, Lacroix R, Bauer KA, Furie BC, Furie B: **Tumor-derived tissue factor-bearing microparticles are associated with venous thromboembolic events in malignancy.** *Clin Cancer Res* 2009, **15:** 6830-6840.
