# Supplementary material for: Circulating microparticles: square the circle
Source: BMC Cell Biol. 2013 Apr 22;14:23. doi: 10.1186/1471-2121-14-23 (PMC3651414; doi:10.1186/1471-2121-14-23)
Supplement: Additional file 7 — References for Table 4 (MP levels in the plasma and body fluids of patients with different disorders). [file 1471-2121-14-23-S7.doc]

**Supplemental file 7.** References for Table 4 (MPs levels in the plasma and body fluids of patients with different disorders).

Agouni A, Lagrue-Lak-Hal AH, Ducluzeau PH, Mostefai HA, Draunet-Bisson C, Leftheriotis G, Heymes C, Martinez MC, Andriantsithaina R: **Endothelial disfunction caused by circulating microparticles from patients with metabolic syndrome.** *Am J Pathol* 2008, **173:** 1210-1219.

Al-Massarani G, Vacher-Coponat H, Paul P, Arnaud L, Loundou A, Robert S, Moal V, Berland Y, Dignat-George F, Camoin-Jau L: **Kidney transplantation decreases the level and procoagulant activity of circulating microparticles.** *Am J Transplant* 2009, **9:** 550-557.

Alijotas-Reig J, Palacio-Garcia C, Farran-Codina I, Ruiz-Romance M, Llurba E, Vilardell-Tarres M: **Circulating cell-derived microparticles in severe preeclampsia and in fetal growth restriction.** *Am J Reprod Immunol* 2012, **67**: 140-151.

Amabile N, Guerin AP, Leroyer A, Mallat Z, Nguyen C, Boddaert J, London GM, Tedgui A, Boulanger CM: **Circulating microparticles are associated with vascular dysfunction in patients with end-stage renal failure.** *J Am Soc Nephrol* 2005, **16:** 3381-3388.

Amabile N, Heiss C, Real WM, Minasi P, McGlothlin D, Rame EJ, Grossman W, De Marco T, Yeqhiazarians Y: **Circulating endothelial microparticle levels predict hemodynamic severity of pulmonary hypertension**. *Am J Respir Crit Care Med* 2008, **177**: 1268-1275.

Amabile N, Heiss C, Chang V, Angeli FS, Damon L, Rame EJ, McGlothlin D, Grossman W, De Marco T, Yeghiazarians Y: **Increased CD62e^+^ endothelial microparticles levels predict poor outcome in pulmonary hypertension patients.** *J Heart Lung Transplant* 2009, **28:** 1081-1086.

Amabile N, Boulanger CM: **Circulating microparticle levels in patients with coronary artery disease, a new indicator of vulnerability?** *Eur Heart J* 2011, **32**: 1958-1960.

Andoh A, Tsujikawa T, Hata K, Araki Y, Kitoh K, Sasaki M, Yoshida T, Fujiama Y: **Elevated circulating platelet-derived microparticles in patients with active inflammatory bowel disease.** *Am J Gastroenterol* 2005, **100:** 2042-2048.

Antwi-Baffour S, Kholia S, Aryee YK-D, Ansa-Addo EA, Stratton D, Lange S, Inal JM: **Human plasma membrane-derived vesicles inhibit the phagocytosis of apoptotic cells – possible role of SLE.** *Biochem Biophys Res Commun* 2010, **398:** 278-283.

Arteaga RB, Chirinos JA, Soriano AO, Jy W, Horstaman L, Jimenez JJ, Mendez AA, Ferreira A, de Marchena E, Ahn YS: **Endothelial microparticles and platelet and leukocyte activation in patients with the metabolic syndrome.** *Am J Cardiol* 2006, **98:** 70-74.

Azzam H, Zagloul M: **Elevated platelet microparticle levels in valvular atrial fibrillation.** *Hematology* 2009, **14**: 357-360.

Baka Z, Senolt L, Vencovsky J, Mann H, Simon PS, Kittel A, Buzas E, Nagy G: **Increased serum concentration of immune cell derived microparticles in polymyositis/dermatomyositis.** *Immunol Lett* 2010, **128:** 124-130.

Bal L, Elderhy S, Di Angelantonio E, Toti F, Zobiari F, Dufaitre G, Meuleman C, Mallat Z, Boccara F, Tedgui A, Freyssinet JM, Cohen A: **Circulating procoagulant microparticles in acute pulmonary embolism: a case-control study.** *Int J Cardiol* **145**: 321-322.

Berckmans RJ, Niewland R, Tak PP, Boeing AN, Romijn FP, Kraan MC, Breedveld FC, Hack CE, Sturk A: **Cell-derived microparticles in synovial fluid from inflamed arthritic joints support coagulation exclusively via a factor VII-dependent mechanism.** *Arthritis Rheum* 2002, **46:** 2857-2866.

Bernal-Mizrachi L, Jy W, Jimenez JJ, Pastor J, Mauro LM, Horstman LL, de Marchena E, Ahn YS: **High levels of circulating endothelial microparticles in patients with acute coronary syndromes.** *Am Heart J* 2003, **145**: 962-970.

Bernard S, Loffroy R, Serusclat A, Boussel L, Bonnefoy E, Thevenon C, Rabilloud M, Revel D, Moulin P, Douek P: **Increased levels of endothelial microparticles CD144 (VE-cadherin) positives in type 2 diabetic patients with coronary noncalcified plaques evaluated by multidetector computed tomography (MDCT).** *Atherosclerosis* 2009, **203**: 429-435

Biasucci LM, Porto I, Di Vito L, de Maria GL, Leone AM, Tinelli G, Tritarelli A, Rocco GD, Snider F, Capogrossi MC, Crea F: **Differences in microparticle release in patients with acute coronary syndrome and stable angina**. *Circ J* 2012, 76: 2174-2182.

Biro E, Nieuwland R, Tak PP, Pronk LM, Schaap MC, Sturk A, Hack CE: **Activated complement components and complement activator molecules on the surface of cell-derived microparticles in patients with rheumatoid arthritis and healthy individuals.** *Ann Rheum Dis* 2007, **66:** 1085-1092.

Boilard E, Nigrovic PA, Larabee K, Watts GF, Coblyn JS, Weinblatt ME, Massarotti EM, Remold-O'Donnell E, Farndale RW, Ware J, Lee DM: **Platelets amplify inflammation in arthritis via collagen-dependent microparticle production.** *Science* 2010, **327:** 580-583.

Bretelle F, Sabatier F, Desprez D, Camoin L, Grunebaum L, Combes V, D'Ercole C, Dignat-George F: **Circulating microparticles: a marker of procoagulant state in normal pregnancy and pregnancy complicated by preeclampsia or intrauterine growth restriction.** *Thromb Haemost* 2003*,* **89**: 486-492.

Brodsky SV, Faccuito ME, Heydt D, Chen J, Islam HK, Kajstura M, Ramaswamy G, Aguero-Rosenfeld M: **Dynamics of circulating microparticles in liver transplant patients.** *J Gastrointestin Liver Dis* 2008, **17:** 261-268.

Brogan PA, Shah V, Brachet, Harnden A, Mant D, Klein N, Dillon MJ: **Endothelial and platelet microparticles in vasculitis of the young.** *Arthritis Rheum* 2004, **50:** 927-936.

Campos FM, Franklin BS, Teixera-Caravalho A, Filho AL, de Paula SC, Fontes CJ, Brito CF, Carvalho LH: **Augmented plasma microparticles during acute *Plasmodium vivax* infection.** *Malar J* 2010, **9:** 327.

Carp H, Dardik R, Lubetsky A, Salomon O, Eskaraev R, Rosenthal E, Inbal A: **Prevalence of circulating procoagulant microparticles in women with recurrent miscarriage: a case-controlled study**. *Hum Reprod* 2004, **19**: 191-195.

Castaman G, Yu-Feng L, Rodeghiero F: **A bleeding disorder characterized by isolated deficiency of platelet microvesicle generation.** *Lancet* 1996, **347:**700-701.

Chahed S, Leroyer AS, Benzerroug M, Gaucher D, Georquescu A, Picaud S, Silvestre JS, Gaudric A, Tedgui A, Massin P, Boulanger CM: **Increased vitreous shedding of microparticles in proliferative diabetic retinopathy stimulates endothelial proliferation.** *Diabetes* 2010, **59:** 694-701.

Chaichompoo P, Kumaya P, Khowawisetsut L, Chiangjong W, Chaiyarit S, Pongsakul N, Sirithenaratanakul N, Fucharoen S, Thongboonkerd V, Pattanapanyasat K: **Characterizations and proteome analysis of platelet-free derived microparticles in β-thalassemia/hemoglobin E patients.** *J Proteomics* 2012, **76** spec No: 239-250. doi: 10.1016/j.jprot.2012.06.004.

Chamouard P, Desprez D, Hugel B, Kunzelmann C, Gidon-Jeangirard C, Lessard M, Baumann R, Freyssinet JM, Grunebaum L: **Circulating cell-derived microparticles in Crohn's disease.** *Dig Dis Sci* 2005, **50:** 574-580.

Chirinos JA, Heresi GA, Velasquez H, Jiminez JJ, Ahn E, Horstman LL, Soriano AO, Zambrano JP, Ahn YS: **Elevation of endothelial microparticles, platelets, and leukocyte activation in patients with venous thromboembolism.** *J Am Coll Cardiol* 2005, **45**: 1467-1471.

Chironi G, Simon A, Hugel B, Del Pino MK, Gariepy J, Freyssinet JM, Tedgui A: **Circulating leukocyte-derived microparticles predict subclinical atherosclerosis burden in asymptomatic subjects.** *Arterioscler Thromb Vasc Biol* 2006, **26:** 2775-2780.

Choudhury A, Chung I, Blann AD, Lip GY: **Elevated platelet microparticles levels in nonvalvular atrial fibrillation: relationship to p-selectin and antithrombotic therapy.** *Chest* 2007, **131**: 809-815.

Combes V, Simon AC, Grau GE, Arnoux D, Camoin L, Sabatier F, Mutin M, Sanmarco M, Sampol J, Dignat-George F: **In vitro generation of endothelial microparticles and possible prothrombotic activity in patients with lupus anticoagulant.** *J Clin Invest* 1999, **104:** 93-102.

Combes V, Taylor TE, Juhan-Vague I, Mege JL, Mwenechanya J, Tembo M, Grau GE, Molyneux ME: **Circulating endothelial microparticles in malawian children with severe falciparum malaria complicated with coma.** *JAMA* 2004, **291:** 2542-2544.

Corrales-Medina VF, Simkins J, Chirinos JA, Serpa JA, Horstman LL, Jy W, Ahn YS: **Increased levels of platelet microparticles in HIV-infected patients with good response to highly active antiretroviral therapy.** *J Acquir Immune Defic Syndr* 2010, **54:** 217-218.

Da Silva EF, Fonseca FA, Franca CN, Ferreira PR, Izar MC, Salomao R, Camargo LM, Tenore SB, Lewi DS: **Imbalance between endothelial progenitors cells and microparticles in HIV-infected patients naive for retroviral therapy.** *AIDS* 2011, **25:** 1595-1601.

Darnige L, Helley D, Fischer AM, Emmerich J, Smadja DM, Fiessinger JN: **Platelet microparticle levels: a biomarker of thromboangiitis obliterans (Buerger’s disease) exacerbation.** *J Cell Mol Med* 2010, **14** (1-2): 449-451.

Daniel L, Fakhouri F, Joly D, Mouthon L, Nusbaum P, Grunfeld JP, Schifferli J, Guillevin L, Lesavre P, Halbwachs-Mecarelli L: **Increase of circulating neutrophil and platelet microparticles during acute vasculitis and hemodialysis.** *Kidney Int* 2006, **69**: 1416-1423.

De Rop C, Stadler M, Buchholz S, Eisert R, Ganser A, Trummer A: **Evaluation of tissue factor bearing microparticles as biomarkers in allogeneic stem-cell transplantation.** *Transplantation* 2011, **92:** 351-358.

Diamant M, Nieuwland R, Pablo RF, Sturk A, Smit JW, Radder JK: **Elevated numbers of tissue-factor exposing microparticles correlate with components of the metabolic syndrome in uncomplicated type 2 diabetes mellitus.** *Circulation* 2002, **106:** 2442-2447.

Dignat-George F, Camoin-Jau L, Sabatier F, Arnoux D, Anfosso F, Bardin N, Veit V, Combes V, Gentile S, Moal V, Sanmarco M, Sampol J: **Endothelial microparticles: a potential contribution to the thrombotic complications of the antiphospholipid syndrome.** *Thromb Haemost* 2004, **91:** 667-673.

Distler JH, Huber LC, Gay S, Distler O, Pisetsky DS: **Microparticles as mediators of cellular cross-talk in inflammatory disease.** *Autoimmunity* 2006, **39:** 683-690.

Dursun I, Duesunsel R, Poyrazoglu HM, Gunduz Z, Patiroglu T, Ulger H, Gurgoze MK: **Circulating endothelial microparticles in children with Henoch-Schonlein purpura: preliminary results.** *Rheumatol Int* 2011, **31**:1595-1600.

Dymicka-Piekarska V, Kemona H, Butkiewicz A, Bychowski J: **Platelets and platelet microparticles glycoprotein IIb/IIIa complex in patients with unstable angina.** *Pol Merkur Lekarski (Pol)* 2005, **18**: 9-12.

Erdbruegger U, Grossheim M, Hertel B, Wyss K, Kirsch T, Woywodt A, Haller H, Haubitz M: **Diagnostic role of endothelial microparticles in vasculitis.** *Rheumatology (Oxford)* 2008, **47:** 1820-1825.

Faure V, Dou L, Sabatier F, Cerini C, Sampol J, Berland Y, Brunet P, Dignat-George F: **Elevation of circulating endothelial microparticles in patients with chronic renal failure.** *J Thromb Haemost* 2006, **4:** 566-573.

Fink K, Feldbruegge L, Schwarz M, Bourgeois N, Helbing T, Bode C, Schwab T, Busch HJ: **Circulating annexin V positive microparticles in patients after successful cardiopulmonary resuscitation**. *Crit Care* 2011, **15**: R251.

Fujimi S, Ogura H, Tanaka H, Koh T, Hosotsubo H, Nakamori Y, Kuwagata Y, Shimazu T, Sugimoto H: **Production of leukocyte microparticles with enhanced expression of adhesion molecules from activated polymorphonuclear leukocytes in severely injured patients.** *J Trauma* 2003, **54**: 114-119.

Galli M, Grassi A, Barbui T: **Platelet-derived microvesicles in thrombotic thrombocytopenic purpura and hemolytic uremic syndrome**. *Thromb Haemost* 1996, **75**: 427-431.

Gao C, Xie R, Yu C, Wang Q, Shi F, Yao C, Xie R, Zhou J, Gilbert GE, Shi J: **Procoagulant activity of erythrocytes and platelets through phosphatidylserine exposure and microparticles release in patients with nephrotic syndrome.** *Thromb Haemost* 2012, **107**: 681-689.

Ge S, Hertel B, Emden SH, Beneke J, Menne J, Haller H, von Vietingoff A: **Microparticle generation and leukocyte death in Shiga toxin-mediated HUS**. *Nephrol Dial Transplant* 2012, **27**: 2768-2775.

Gelderman MP, Schiffmann R, Simak J: **Elevated endothelial microparticles in Fabry children decreased after enzyme replacement therapy**. *Arterioscler Thromb Vasc Biol* 2007, **27**: e138-e139.

Gemmel CH, Sefton MV, Yeo EL: **Platelet-derived microparticle formation involves glycoprotein IIb-IIIa. Inhibition by RGDS and a Glanzmann’s thromboasthenia defect.** *J Biolog Chem* 1993, **268**: 14586-14589.

Georgescu A, Alexandru N, Popov D, Amuzescu M, Andrei E, Zamfir C, Maniu H, Badila A: **Chronic venous insufficiency is associated with elevated level of circulating microparticles**. *J Thromb Haemost* 2009, **7**:1566-1575.

Gerotziafas GT, Van Dreden P, Chaari M, Galea V, Khaterchi A, Lionnet F, Stankovic-Stojanovic K, Blanc-Brude O, Woodhams B, Maier-Redelsperger M, Girot R, Hatmi M, Elalamy I: **The acceleration of the propagation phase of thrombin generation in patients with steady-state sickle cell disease is associated with circulating erythrocyte-derived microparticles.** *Thromb Haemost* 2012, **107**: 1044-1052.

Goswami D, Tannetta DS, Magee LA, Fuchisawa A, Redman CW, Sargent IL, von Dadelszen P: **Excess syncytiotrophoblast microparticle shedding is a feature of early onset pre-eclampsia, but not normotensive intrauterine growth restriction.** *Placenta* 2006, **27:** 56-61.

Gris JC, Toulon P, Brun S, Maugard C, Sarlat C, Schved JF, Berlan J: **The relationship between plasma microparticles, protein S and cardiolipin antibodies in patients with human immjunodeficiency virus infection.** *Thromb Haemost* 1996, **76:** 38-45.

Guervilly C, Lacroix R, Forel JM, Roch A, Camoin-Jau L, Papazian L, Diqnat-George F: **High levels of circulating leukocyte microparticles are associated with better outcome in acute respiratory distress syndrome.** *Crit Care* 2011, **15:** R31.

Guiducci S, Distler JH, Juengel A, Huscher LC, Huber LC, Michel BA, Gay RE, Pisetsky DS, Gay S, Matucci-Cerinic M, Distler O: **The relationship between plasma microparticles and disease manifestations in patients with systemic sclerosis.** *Arthritis Rheum* 2008, **58:** 2845-2853.

Guiducci S, Ricci L, Romano E, Ceccarelli C, Distler JH, Miniati I, Calabri GB, Distler O, Matucci Cerinic M, Falcini F: **Microparticles and Kawasaki disease: a marker of vascular damage?** *Clin Exp Rheumatol* 2011, **29**: S121-S125.

Habib A, Kunzelmann C, Shamseddeen W, Zobairi F, Freyssinet JM, Taher A: **Elevated levels of circulating procoagulant microparticles in patients with beta-thalassemia intermedia.** *Haematologica* 2008, **93:** 941-942.

Helal O, Defoort C, Robert S, Marin C, Lesavre N, Lopez-Miranda J, Riserus U, Basu S, Lovegrove J, McMonagle J, Roche HM, Dignat-George F, Lairon D: **Increased levels of microparticles originating from endothelial cells, platelets and erythrocytes in subjects with metabolic syndrome: relationship with oxidative cells.** *Nutrition, Metabolism and Cardiovasc Dis* 2011, **21:** 665-671.

Helley D, de Latour RP, Porcher R, Rodrigues CA, Galy-Fauroux I, Matheron J, Duval A, Schved JF, Fisher AM, et al.: **Evaluation of hemostasis and endothelial function in patients with paroxysmal nocturnal hemoglobinuria receiving eculizumab.** *Haematologica* 2010, **95**: 574-581.

Holme PA, Mueller F, Solum NO, Brosstad F, Froeland SS, Aukrust P: **Enhanced activation of platelets with abnormal release of RANTES in human immunodeficiency virus type 1 infection.** *FASEB J* 1998, **12:** 79-89.

Huang PH, Huang SS, Chen YH, Lin CP, Chiang KH, Chen JS, Tsai HY, Lin FY, Chen JW, Lin SJ: **Increased circulating CD31+/annexin V+ apoptotic microparticles and decreased circulating endothelial progenitor cell levels in hypertensive patients with microalbuminuria.** *J Hypertens* 2010, **28**: 1655-1665.

Hugel B, Socie G, Vu T, Toti F, Gluckman E, Freyssinet JM, Scrobohaci ML: **Elevated levels of circulating procoagulant microparticles in patients with paroxysmal nocturnal hemoglobinuria and aplastic anemia.** *Blood* 1999, **93:** 3451-3456.

Jiminez JJ, Jy W, Mauro LM, Horstman LL, Ahn YS: **Elevated endothelial microparticles in thrombotic thrombocytopenic purpura: findings from brain and renal microvascular cell culture and patients with active disease.** *Br J Haematol* 2001, **112:** 81-90.

Jimenez J, Jy W, Mauro LM, Horstman LL, Ahn ER, Ahn YS, Minagar A: **Elevated endothelial microparticle-monocyte complexes induced by multiple sclerosis plasma and the inhibitory effects of interferon-beta 1b on release of endothelial microparticles, formation and transendothelial migration of monocyte-endothelial microparticle complexes**. *Mult Scler* 2005, **11**: 310-315.

Joop K, Berckmans RJ, Nieuwland R, Berkhout J, Romijn FP, Hack CE, Sturk A: **Microparticles from patients with multiple organ disfunction syndrome and sepsis support coagulation through multiple mechanisms.** *Thromb Haemost* 2001, **85:** 810-820.

Joseph JE, Harrison P, Mackie IJ, Isenberg DA, Machin SJ: **Increased circulating platelet-leukocyte complexes and platelet activation in patients with antiphospholipid syndrome, systemic lupus erythematosus and rheumatoid arthritis.** *Br J Haematol* 2001, **115:** 451-459.

Jung KH, Chu K, Lee ST, Bahn JJ, Kim JH, Kim M, Lee SK, Roh JK: **Risk of macrovascular complications in type 2 diabetes mellitus: endothelial microparticles profiles.** *Cerebrovasc Dis* 2009a, **31:** 485-493.

Jung KH, Chu K, Lee ST, Park HK, Bahn JJ, Kim DH, Kim JH, Kim M, Kun Lee S, Roh JK: **Circulating endotelial microparticles as a marker of cerebrovascular disease.** *Ann Neurol* 2009b, **66:** 191-199.

Jy W, Horstman LL, Arce M, Ahn YS: **Clinical significance of platelet microparticles in autoimmune thrombocytopenia.** *J Lab Clin Med* 1992, **119**:334-345.

Jy W, Horstmann LL, Jimenez JJ, Ahn JS: **Measuring circulating cell-derived microparticles**. *J Thromb Haemost* 2004a, **2:** 1842-1843.

Jy W, Minagar A, Jimenez JJ, Sheremata WA, Mauro LM, Horstman LL, Bidot C, Ahn YS: **Endothelial microparticles (EMP) bind and activate monocytes: elevated EMP-monocyte conjugates in multiple sclerosis.** *Front Biosci* 2004b, **9:** 3137-3144.

Jy W, Tiede M, Bidot CJ, Horstman LL, Jimenez JJ, Chirinos J, Ahn YS: **Platelet activation rather than endothelial injury identifies risk of thrombosis in subjects positive for antiphospholipid antibodies.** *Thromb Haemost* 2007, **121:** 319-325.

Kim SJ, Moon GJ, Cho YH, Kang HY, Hyung NK, Kim D, Lee JH, Nam JY, Bang OY. **Circulating mesenchymal stem cells microparticles in patients with cerebrovascular disease.** *PLoS One* 2012, **7**: e37036.

Knijff-Dutmer EA, Koerts J, Nieuwland R, Kalsbeek-Batenburg EM, van de Laar MA: **Elevated levels of micropartyicles are associated with disease activity in rheumatoid arthritis.** *Arthritis Rheum* 2002, **46:** 1498-1503.

Koga H, Sugiyama S, Kugiyama K, Watanabe K, Fukushima H,, Tanaka T, Sakamoto T, Yoshimura M, Jinnouchi H, Ogawa H: **Elevated levels of VE-cadherin-positive endothelial microparticles in patients with type 2 diabetes mellitus and coronary artery disease.** *J Am Coll Cardiol* 2005, **45:** 1622-1630.

Koga H, Sugiyama S, Kugiyama K, Fukushima H, Watanabe K, Sakamoto T, Yoshimura M, Jinnouchi H, Ogawa H: **Elevated levels of remnant lipoproteins are associated with plasma platelet microparticles in patients with type-2 diabetes mellitus without obstructive coronary artery disease.** *Eur Heart J* 2006, **27:** 817-823.

Koiou E, Tziomalos K, Katsikis I, Kalaitzakis E, Kandaraki EA, Tsourdi EA, Delkos D, Papadakis E, Panidis D: **Circulating platelet-derived microparticles are elevated in women with polycystic ovary syndrome diagnosed with the 1990 criteria and correlate with serum testosterone levels.** *Eur J Endocrinol* 2011, **165:** 63-68.

Koiou E, Tziomalos K, Katsikis I, Papadakis E, Kandaraki EA, Panidis D: **Platelet-derived microparticles in overweight/obese women with the polycystic ovary syndrome.** *Gynecol Endocrinol* 2013, **29**: 250-253.

Kornek M, Popov Y, Libermann TA, Afdahl NH, Schupann D: **Human T cell microparticles circulate in blood of hepatitis patients and induce fibrolytic activation of hepatic stellate cells.** *Hepatology* 2011, **53:** 230-242.

Kornek M, Lynch M, Mehta SH, Lai M, Exley M, Afdahl NH, Schuppan D. **Circulating microparticles as disease-specific biomarkers of severity of inflammation in patients with hepatitis C or nonalcoholic steatohepatitis.** *Gastroenterology* 2012, **143**: 448-458.

Kuempers P, Erdbruegger U, Grossheim M, Meyer GP, Hiss M, Gwinner W, Haller H, Haubitz M: **Endothelial microparticles as a diagnostic aid in Churg-Strauss vasculitis-induced cardiomyopathy.** *Clin Exp Rheumatol* 2008, **26**: S86-S89.

Kuriyama N, Nagakane Y, Hosomi A, Ohara T, Harada S, Takeda K, Yamada K, Ozasa K, Tokuda T, Watanabe Y, Mizuno T, Nakagawa M: **Evaluation of factors associated with elevated levels of platelet-derived microparticles in the acute phase of cerebral infarction.** *Clin Appl Thromb Hemost* 2010, **16**: 26-32.

Lackner P, Dietmann A, Beer R, Fischer M, Broessner G, Helbok R, Marxgut J, Pfausler B, Schmutzhard E: **Cellular microparticles as a marker for cerebral vasospasm in spontaneous subarachnoid hemorrhage.** *Stroke* 2010, **41:** 2353-2357.

Larkin M: **Raised endothelial microparticles: an early marker for multiple sclerosis?** *Lancet* 2001, **357**: 1679.

Lee YJ, Jy W, Horstman LL, Janania J, Reyes Y, Kelley RE, Ahn YS: **Elevated platelet microparticles in transient ischemic attacks, lacunar infarcts, and multiinfarct dementias.** *Thromb Res* 1993, **72:** 295-304.

Leroyer AS, Tedgui A, Boulanger CM: **Microparticles and type 2 diabetes.** *Diab Metab* 2008, **34:** 27-31.

Liebman HA, Feinstein DI: **Thrombosis in patients with paroxysmal nocturnal hemoglobinuria is associated with markedly elevated plasma levels of leukocyte-derived tissue factor.** *Thromb Res* 2003, **111:** 235-238.

Lok CA, Van Der Post JA, Sargent IL, Hau CM, Sturk A, Boer K, Nieuwland R: **Changes in microparticle numbers and cellular origin during pregnancy and preeclampsia.** *Hypertens Pregnancy* 2008, **27:** 344-360.

Lok CA, Jebbink J, Nieuwland R, Faas MM, Boer K, Sturk A, Van Der Post JA: **Leukocyte activation and circulating leukocyte-derived microparticles in preeclampsia.** *Am J Reprod Immunol* 2009, **61**: 346-359.

Macey M, Hagi-Pavli E, Stewart J, Wallace GR, Stanford M, Shirlaw P, Fortune F: **Age, gender and disease-related platelet and neutrophil activation ex vivo in whole blood samples from patient's with Behcet's disease.** *Rheumatology* 2011, **50**:1849-1859.

Mallat Z, Benamer H, Hugel B, Benessiano J, Sterg PG, Freyssinet JM, Tedgui A: **Elevated levels of shed microparticles with procoagulant potential in the peripheral circulating blood of patients with acute coronary syndromes.** *Circulation* 2001, **101:** 841-843.

Maruyama K, Morishita E, Sekiya A, Omote M, Kadono T, Asakura H, Hashimoto M, Kobayashi M, Nakatsumi Y, Takada S, Ohtake S: **Plasma levels of platelet-derived microparticles in patients with obstructive sleep apnea syndrome.** *J Atheroscler Thromb* 2012, **19**: 98-104.

Mayne E, Funderburg NT, Sieg SF, Asaad R, Jiang W, Kalinowska M, Luciano AA, Stevens W, Rodriguez B, Brenchley JM, Douek DC, Lederman MM: **Increased platelet and microparticle activation in HIV infection: upregulation of P-selectin and tissue factor expression.** *J Acquir Immune Defic Syndr* 2011, **59**: 340-346.

Merino A, Portoles J, Selgas R, Oieda R, Buendia P, Ocana J, Baio MA, del Peso G, Carracedo J, Ramirez R, Martin-Malo A, Aljama P: **Effect of different dialysis on microinflammatory status and endothelial damage.** *Clin J Am Soc Nephrol* 2010, **5:** 227-234.

Messer L, Alsaleh G, Freyssinet JM, Zobairi F, Leray I, Gottenberg JE, Sibilia J, Toti-Orfanoudakis F, Wachsmann D: **Microparticle-induced release of B-lymphocyte regulators by rheumatoid synoviocytes.** *Arthritis Res Ther* 2009, **11:** R40.

Michelsen AE, Noto AT, Brodin E, Mathiensen EB, Brosstad F, Hansen JB: **Elevated levels of platelet microparticles in carotid atherosclerosis and during the postprandial state.** *Thromb Res* 2009, **123:** 881-886.

Minagar A, Jy W, Jimenez JJ, Sheremata WA, Mauro LM, Mao WW, Horstman LL, Ahn YS: **Elevated plasma endothelial microparticles in multiple sclerosis.** *Neurology* 2001, **56:** 1319-1324.

Mobarrez F, Nybom R, Johansson V, Hultman CM, Wallen H, Landen M, Wetterberg L: **Microparticles and microscopic structures in three fractions of fresh cerebrospinal fluid in schizophrenia: case report of twins**. *Schizophr Res* 2013, **143**: 192-197.

Morel O, Jesel L, Freyssinet JM, Toti F: **Elevated levels of procoagulant microparticles in a patient with myocardial infarction, antiphospholipid antibodies and multifocal cardiac thrombosis.** *Thromb J* 2005, **3:** 15.

Mostefai HA, Meziani F, Mastronardi ML, Agouni A, Heymes C, Sargentini C, Asfar P, Martinez MC, Andriantsitohaina R: **Circulating microparticles from patients with septic shock exert protective role in vascular function.** *Am J Resp Crit Care Med* 2008, **178:** 1148-1155.

Nagahama M, Nomura S, Ozaki Y, Yoshimira C, Kagawa H, Fukuhura S: **Platelet activation markers and soluble adhesion molecules in patients with systemic lupus erythematosus.** *Autoimmunity* 2001, **33:** 85-94.

Nantakomol D, Dondorp AM, Krudsood S, Udomsangpetch R, Pattanapanyasat K, Combes V, Grau GE, White NJ, Viriyavejakul P, Day NP, Chotivanich K: **Circulating red cell-derived microparticles in human malaria.** *J Infect Dis* 2011, **203:** 700-706.

Nielsen CT, Ostergaard O, Johnsen C, Jacobsen S, Heegaard NH: **Distinct features of circulating microparticles and their relationship to clinical manifestations in systemic lupus erythematosus**. *Arthritis Rheum* 2011; **63**: 3067-3077.

Nielsen CT, Ostergaard O, Stener L, Iversen LV, Truedsson L, Gullstrand B, Jacobsen S, Heegaard NH: **Increased IgG on cell-derived plasma microparticles in systemic lupus erythematosus is associated with autoantibodies and complement activation.** *Arthritis Rheum* 2012, **64**: 1227-1236.

Nieuwland R, Berckmans RJ, McGregor S, Boeing AN, Romijn FP, Westendorp RG, Hack CE, Sturk A: **Cellular origin and procoagulant properties of microparticles in meningococcal sepsis.** *Blood* 2000, **95:** 930-935.

Nomura S, Shouzu A, Nishikawa M, Kokawa T, Yasunaga K: **Significance of platelet-derived microparticles in uremia.** *Nephron* 1993, **63:** 485.

Nomura S, Suzuki M, Katsura K, Xie GL, Miyazaki Y, Miyake T, Kido H, Kagawa H, Fukuhara S: **Platelet-derived microparticles may influence the development of atherosclerosis in diabetes mellitus.** *Atherosclerosis* 1995, **116:** 235-40.

Nomura S, Inami N, Iwasaka T, Liu Y: **Platelet activation markers, microparticles and soluble adhesion molecules are elevated in patients with arteriosclerosis obliterans: therapeutic effects by cliostazol and potentiation by dipyramidole.** *Platelets* 2004a, **15:** 167-172.

Nomura S, Ishii K, Kanazawa S, Inami N, Uoshima N, Ishida H, Yoshinara T, Kitayama H, Hayashi K:. **Significance of elevation in cell-derived microparticles after allogeneic stem cell transplantation: transient elevation of platelet-derived microparticles in TMA/TTP.** *Bone Marrow Transplant* 2005; **36:**921-922.

Nomura S, Ishii K, Inami N, Kimura Y, Uoshima N, Ishida H, Yoshinara T, Urase F, Maeda Y, Hayashi K: **Evaluation of angiopoietins and cell-derived microparticles after stem cell transplantation.** *Biol Blood Marrow Transplant* 2008, **14:** 766-774.

Nomura S, Shouzu A, Omoto S, Nishikawa M, Iwasaka T, Fukuhara S: **Activated platelet and oxidized LDL induce endothelial membrane vesiculation: clinical significance of endothelial cell-derived microparticles in patients with type 2 diabetes.** *Clin Appl Thromb Hemost* 2004b, **10:** 205-215.

Nomura S, Inami N, Ozaki Y, Kagawa H, Fukuhura S: **Significance of microparticles in progressive systemic sclerosis with interstitial pneumonia.** *Platelets* 2008, **19**: 192-198.

Nomura S: **Dynamic role of microparticles in type 2 diabetus mellitus.** *Curr Diabetes Rev* 2009, **5:** 245-251.

Nomura S, Shouzu A, Omoto S, Inami N, Ueba T, Urase F, Maeda Y: **Effects of eicosapentaenoic acid on endothelial cell–derived microparticles, angiopoietins and adiponectin in patients with type 2 diabetus**. *J Atheroscler Thromb* 2009, **16**: 83-90.

Nomura S, Omoto S, Yokoi T, Fujita S, Ozasa R, Eguchi N, Shouzu A: **Effects of miglitol in platelet-derived microparticle, adiponectin, and selectin level in patients with type 2 diabetes mellitus**. *Int J Gen Med* 2011, **4:**539-545.

Oehmcke S, Moergelin M, Malmstroem J, Linder A, Chew M, Thorlacius H, Herwald H: **Stimulation of blood mononuclear cells with bacterial virulence factors leads to the release of pro-coagulant and pro-inflammatory microparticles.** *Cell Microbiol* 2012, 14:107-119.

Ogata N, Imaizumi M, Nomura S, Shozu A, Arichi M, Matsuoka M, Matsumura M: **Increased levels of platelet-derived microparticles in patients with diabetic retinopathy.** *Diabetes Res Clin Pract 2005*, **68:** 193-201.

Ogata N, Nomura S, Shouzu A, Imazuimi M, Arichi M, Matsumura M: **Elevation of monocyte-derived microparticles in patients with diabetic retinopathy.** *Diabetes Res Clin Pract* 2006, **73:** 241-248.

Ogura H, Kawasaki T, Tanaka H, Koh T, Tanaka R, Ozeki Y, Hosotsubo H, Kuwagata Y, Shimazu T, Sugimoto H: **Activated platelets enhance microparticle formation and platelet-leukocyte interaction in severe trauma and sepsis.** *J Trauma* 2001, **50:** 801-809.

Ogura H, Tanaka H, Koh T, Fujita K, Fujimi S, Nakamori Y, Hosotsubo H, Kuwagata Y, Shimazu T, Sugimoto H: **Enhanced production of endothelial microparticles with increased binding to leukocytes in patients with severe systemic inflammatory response syndrome.** *J Trauma* 2004, **56:** 823-830.

Oyabu C, Morinobu A, Sugiama D, Saegusa J, Tanaka S, Morinobu S, Tsuji G, Kasagi S, Kawano S, Kumagai S: **Plasma platelet-derived microparticles in patients with connective diseases.** *J Rheumatol* 2011, **38:** 680-684.

Palkovits J, Novacek G, Kollars M, Hron G, Osterode W, Quehenbergher P, Kyrle PA, Vogelsang H, Reinish W, Papay P, Weltermann A: **Tissue factor exposing microparticles in inflammatory bowel disease.** *J Crohns Colitis* 2013, 7:222-229.

Pankoui Mfonkeu JB, Gouado I, Fotso Kuate H, Zambou O, Amvam Zoll PH, Grau GE, Combes V: **Elevated cell-specific microparticles are a biological marker for cerebral dysfunctions in human severe malaria**. *PLoS One* 2010, **5**: e13415.

Park MS, Owen BA, Ballinger BA, Sarr MG, Schiller HJ, Zietlow SP, Jenkins DH, Ereth MH, Owen WG, Heit JA: **Quantification of hypercoagulable state after blunt trauma: microparticle and thrombin generation are increased relative to injury severity, while standard markers are not**. *Surgery* 2012, **151**:831-836.

Pattanapanyasat K, Noulsri E, Fucharoen S, Lerdwana S, Lamchiagdhase P, Siritanaratkul N, Webster HK: **Flow cytometric quantitation of red blood vesicles in thalassemia.** *Cytometry B* 2004, **57**: 23-31.

Pattanapanyasat K, Gonwong S, Chaichompoo P, Noulsri E, Lerdwana S, Sukariprom K, Siritanaratkui N, Fucharoen S: **Activated platelet-derived microparticles in thalassemia.** *Br J Haematol* 2007; **136:** 462-471.

Pelletier F, Garnache-Ottou F, Angelot F, Biichle S, Vidal C, Humbert P, Saas P, Seilles E, Aubin F: **Increased levels of circulating endothelial-derived microparticles and small-size platelet-derived microparticles in psoriasis.** *J Invest Dermatol* 2011, **131:** 1573-1576.

Pereira J, Alfaro G, Goycoolea M, Quiroga T, Ocguetau M, Massardo L, Perez C, Saez C, Panes O, Matus V, Mezzano D: **Circulating platelet-derived microparticles in systemic lupus erythematosus. Association with increased thrombin generation and procoagulant state.** *Thromb Haemost* 2006, **95**: 94-99.

Perez-Casal M, Thompson V, Downey C, Welters I, Wyncoll D, Thachil J, Toh CH: **The clinical and functional relevance of microparticles induced by activated protein C treatment in sepsis.** *Crit Care* 2011, **15:** R195.

Pihusch R, Wegner H, Salat C, Pihusch M, Holler E, Kolb HJ, Hiller E: **Flow cytometric findings in platelets of patients following hematopoietic stem cell transplantation.** *Bone Marrow Transplant* 2002, **30:** 381-387.

Pirro M, Bocci EB, Di Filippo F, Schillaci G, Mannarino MR, Bagaglia F, Gerli R, Mannarino E: **Imbalance between endothelial injury and repair in patients with polymyalgia rheumatica: improvement with corticosteroid treatment**. *J Intern Med* 2012, **272**: 177-184.

Porro C, Lepore S, Trotta T, Castellani S, Ratclif L, Battaglino A, Di Gioia S, Martinez MC, Conese M, Maffione AB: **Isolation and characterization of microparticles in sputum from cystic fibrosis patients.** *Respir Res* 2010, **11**: 94.

Preston RA, Jy W, Jimenez JJ, Mauro LM, Horstman LL, Valle M, Aime G, Ahn YS: **Effects of severe hypertension on endothelial and platelet microparticles**. *Hypertension* 2003, **41**:211-217.

Proulle V, Hugel B, Guillet B, Grunebaum L, Lambert T, Freyssinet JM, Dreyfus M: **Circulating microparticles are elevated in haemophiliacs and non-haemophilic individuals aged <18 years.** *Br J Haematol* 2005, **131:** 487-489.

Rahman S, Cichon M, Hoppensteadt D, Cunanan J, Jeske W, Thethi I, Fareed J: **Upregulation of microparticles in DIC and its impact on inflammatory processes**. *Clin Appl Thromb Hemost* 2011, 17: E202-E204.

Rank A, Nieuwland R, Toth B, Pihusch V, Hiller E, Kolb HJ, Pihusch R: **Microparticles for diagnosis of graft-versus-host disease after allogeneic stem transplantation.** *Transplantation* 2011, **92:** 244-250.

Rank A, Nieuwland R, Nikolajek K, Roesner S, Wallwiener LM, Hiller E, Toth B: **Hormone replacement therapy leads to increased plasma levels of platelet derived microparticles in postmenopausal women.** *Arch Gynecol Obstet* 2012, **285**:1035-1041.

Rautou PE, Bresson J, Sainte-Marie Y, Vion AC, Paradis V, Renard JM, Devue C, Heymes C, Letteron P, Elkrief L, Lebrec D, Valla D, Tedgui A, Moreau R, Boulanger CM: **Abnormal plasma microparticles impair vasoconstrictor responses in patients with cirrhosis.** *Gastroenterology* 2012, **143**: 166-176.

Reyna-Villasmil E, Mejia-Montilla J, Reyna-Villasmil N, Torres-Cepeda D, Pena-Paredes E, Santos-Bolivar J, Perozo-Romero J: **Endothelial microparticles in preeclampsia and eclampsia.** *Med Clin (Barc)* 2011, **136:** 522-526 (Spanish).

Sabatier F, Darmon P, Hugel B, Combes V, Sanmarco M, Velut JG, Arnoux D, Charpiot P, Freyssinet JM, Oliver C, Sampol J, Dignat-George F: **Type 1 and type 2 diabetic patients display different patterns of cellular microparticles.** *Diabetes* 2002, **51:** 2840-2845

Sari I, Bozkaya G, Kirbiyik H, Alacacioglu A, Ates H, Sop G, Can G, Taylan A, Piskin O, Yildiz Y, Akkoc N: **Evaluation of circulating endothelial and platelet microparticles in men with ankylosing spondylitis.** *J Rheumatol* 2012, **39**: 594-599.

Schmelzle M, Splith K, Andersen LW, Kornek M, Schuppan D, Jones-Bamman C, Nowak M, Toxavidis V, Salhanick SD, Han L, Schulte Esch J, Jonas S, Donnino MW, Robson SC: **Increased plasma levels of microparticles expressing CD39 and CD133 in acute liver injury.** *Transplantation* 2013, **95**: 63-69.

Sellam J, Proulle V, Jungel A, Ittah M, Miceli RC, Gottenberg JE, Toti F, Benessiano J, Gay S, Freyssinet JM, Mariette X: **Increased levels of circulating microparticles in primary Sjogren syndrome, systemic lupus erythematosus and rheumatoid arthritis and relation with disease activity.** *Arthritis Res Ther* 2009, **11:** R156.

Sewify EM, Sayed D, Abdel Aal RF, Ahmad HM, Abdou MA: **Increased red cell microparticles and platelet microparticles in immune thrombocytopenic purpura.** *Thromb Res* 2013, 131: e59-e63.

Sheremata WA, Jy W, Delgado S, Minagar A, McLarthy J, Ahn Y: **Interferon-beta1a reduces plasma CD31^+^ endothelial micropartyicles (CD31^+^EMP) in multiple sclerosis.** *J Neuroinflammation* 2006, **3**:23.

Sheremata WA, Jy W, Horstman LL, Ahn YS, Alexander JS, Minagar A: **Evidence of platelet activation in multiple sclerosis.** *J Neuroinflammation* 2008, **5**:27.

Shet AS, Aras O, Gupta K, Hass MJ, Rausch DJ, Saba N, Koopmeiners L, Key NS, Hebbel RP: **Sickle blood contains tissue factor positive microparticles derived from endothelial cells and monocytes.** *Blood* 2003, **102:** 2678-2683.

Shirafuji T, Hamaguchi H, Higuchi M, Kanda F: **Measurement of platelet-derived microparticle levels using an enzyme-linked immunosorbent assay in polymyositis and dermatomyositis patients**. *Muscle Nerve* 2009, **39**: 586-590.

Shouzu A, Nomura S, Omoto S, Hayakawa T, Nishikawa M, Iwasaka T: **Effect of ticlopidine on monocyte-derived microparticles and activated platelet markers in diabetes mellitus.** *Clin Appl Thromb Hemost* 2004, **10**: 167-173.

Simak J, Holada K, Risitano AM, Zivny JH, Young NS, Vostal JG: **Elevated circulating endothelial membrane microparticles in paroxysmal nocturnal haemoglobinuria.** *Br J Haematol* 2004, **125:** 804-813.

Simak J, Gelderman MP, Yu H, Wright V, Baird AE: **Circulating endothelial microparticles in acute ischemic stroke: a link to severity, lesion volume and outcome.** *J Thromb Haemost* 2006, **4:**1296-1302.

Sims PJ, Wiedmer T, Esmon CT, Weiss HJ, Shattil SJ: **Assembly of the platelet prothrombinase complex is linked to vesiculation of the platelet plasma membrane. Studies in Scott syndrome: an isolated defect in platelet procoagulant activity.** *J Biol Chem* 1989, **264:** 17049-17057.

Singh N, Gemmell CH, Daly PA, Yeo EL: **Elevated platelet-derived microparticles levels during unstable angina.** *Can J Cardiol* 1995, **11:** 1015-1021.

Stahl AL, Sartz L, Nelsson A, Bekassy ZD, Karpmann D: **Shiga-toxin and lipopolysaccharide induce platelet-leukocyte aggregates and tissue factor release, a thrombocytic mechanism in hemolytic uremic syndrome.** *PLoS One* 2009, **4**:e6990.

Stahl AL, Sartz L, Karpman D: **Complement activation on platelet-leukocyte complexes and microparticles in enterohemorrhagic Escherichia coli-induced hemolytic uremic syndrome.** *Blood* 2011, **117:** 5503-5513.

Stepien E, Stankiewicz E, Zalewski J, Godlewski J, Zmudka K, Wybranska I: **Number of microparticles generated during acute myocardial infarction and stable angina correlates with platelet activation**. *Arch Med Res* 2012, **43**: 31-35.

Steppich BA, Hassenpflug M, Braun SL, Schoemig K, von Beckerath O, von Beckerath N, Eckstein HH, Ott I: **Circulating tissue factor and microparticles are not increased in patients with deep vein thrombosis.** *Vasa* 2011, **40:** 117-122.

Tamagawa-Mineoka R, Katoh N, Ueda E, Masuda K, Kishimoto S: **Platelet-derived microparticles and soluble P-selectin as platelet activation markers in patients with atopic dermatitis.** *Clin Immunol* 2009, **131:** 495-500.

Tamagawa-Mineoka R, Katoh N, Kishimoto S: **Platelet activation in patients with psoriasis: increased plasma levels of platelet-derived microparticles and soluble P-selectin.** *J Am Acad Dermatol* 2010, **62:** 621-626.

Tan KT, Tayebjee MH, Lim HS, Lip GY: **Clinically apparent atherosclerotic disease in diabetes is associated with an increase in platelet microparticle levels.** *Diabet Med* 2005, **22:** 1657-1662.

Tan Z, Yuan Y, Chen S, Chen Y, Chen TX: **Elevated plasma endothelial microparticles together with TNF-alpha and IL-6 in Kawasaki disease.** *Indian Pediatr* 2012, Epub Nov 5.

Tantawy AA, Matter RM, Hamed AA, Shams El Din El Telbany MA: **Platelet microparticles in immune thrombocytopenic purpura in pediatrics.** *Pediatr Hematol Oncol* 2010, **27**: 283-296.

Toti F, Satta N, Fressinaud E, Meyer D, Freyssinet JM: **Scott syndrome, characterized by impaired transmembrane migration of procoagulant phosphatidylserine and hemorrhagic complications, is an inherited disorder**. *Blood* 1996, **87**: 1409-1415.

Tramontano AF, Lyubarova R, Tsiakos J, Palaia T, Deleon JR, Ragolia L: **Circulating endothelial microparticles in diabetes mellitus.** *Mediators Inflamm* 2010, 250476, doi: 10.1155/2010/250476.

Trappenburg MC, van Schilfgaarde M, Marchetti M, Spronk HM, ten Cate H, Leyte A, Terpstra WE, Falanga A: **Elevated procoagulant microparticles expressing endothelial and platelet markers in essential thrombocythemia.** *Haematologica* 2009, **94**: 911-918.

Trummer A, de Rop C, Stadler M, Ganser A, Buchholz S: **P-selectin glycoprotein ligand-1 positive microparticles in allogeneic stem cell transplantation of hematologic malignancies.** *Exp Hematol* 2011, **39**: 1047-1055.

Tsimerman G, Roquin A, Bachar A, Melamed E, Brenner B, Aharon A: **Involvement of microparticles in diabetic vascular complications.** *Thromb Haemost* 2011, **106:** 310-321

Ueba T, Haze T, Sugiyama M, Higuchi M, Asayama H, Karitani Y, Nishikawa T, Yamashita K, Nagami S, Nakayama T, Kanatani K, Nomura S**: Level, distribution and correlates of platelet-derived microparticles in healthy individuals with special reference to the metabolic syndrome.** *Thromb Haemost* 2008, **100:** 280-285

Umekita K, Hidaka T, Ueno S, Takajo I, Kai Y, Naqatomo Y, Sawaguchi A, Suganuma T, Okayama A: **Leukocytapheresis (LCAP) decreases the level of platelet-derived microparticles (MPs) and increases the level of granulocytes-derived MPs: a possible connection with the effect of LCAP on rheumatoid arthritis.** *Mod Rheumatol* 2009, **19:** 265-272.

Van Beers EJ, Schaap MC, Berckmans RJ, Niewland R, Sturk A, van Doormaal FF, Meijers JC, Biemond BJ, CURAMA study group: **Circulating erythrocyte-derived microparticles are associated with coagulation activation in sickle disease.** *Haematologica* 2009, **94:** 1513-1519.

Van Eijk, Tushuizen ME, Sturk A, Dijkmans BA, Boers M, Voskuyl AE, Diamant M, Wolbink GJ, Nieuwland R, Nurmohamed MT: **Circulating microparticles remain associated with complement activation despite intensive anti-inflammatory therapy in early rheumatoid arthritis**. *Ann Rheum Dis* 2010, **69**: 1378-1382.

Van Tits LJ, van Heerde WL, Landburg PP, Boderie MJ, Muskiet FA, Jacobs N, Duits AJ, Schnog JB: **Plasma annexin A5 and microparticle phosphatidylserine levels are elevated in sickle cell disease and increase further during painful crisis.** *Biochem Biophys Res Commun* 2009, **390:** 161-164.

VanWijk MJ, Nieuwland R, Boer K, van der Post JA, VanBavel E, Sturk A: **Microparticle subpopulations are increased in preeclampsia: possible involvement in vascular dysfunction?** *Am J Obstet Gynecol* 2002, **187:** 450-456.

Vedder AC, Biro E, Aerts JM, Nieuwland R, Sturk G, Hollak CE: **Plasma markers of coagulation and endothelial activation in Fabry disease: impact of renal impairment.** *Nephrol Dial Transplant* 2009, **24**: 3074-3081.

Vikerfors A, Mobarezz F, Bremme K, Holmstroem M, Agren A, Eelde A, Bruzelius M, Antovic A, Wallen H, Svenungsson E: **Studies of microparticles in patients with the antiphospholipid syndrome (APS).** *Lupus* 2012, **21:** 802-805.

Walenta K, Schwarz V, Schirmer SH, Kindermann I, Friedrich EB, Solomayer EF, Sliwa K, Labidi S, Hilfiker-Kleiner D, Boehm M: **Circulating microparticles as indicators of peripartum cardiomyopathy**. *Eur Heart J* 2012, **33**: 1469-1479.

Werner N, Wassmann S, Ahlers P, Kosiol S, Nickenig G: **Circulating CD31^+^/annexin V^+^ apoptotic microparticles correlate with endothelial function in patients with coronary artery disease.** *Arterioscler Thromb Vasc Biol* 2006, **26:** 112-116.

Woth G, Tokes-Fuezesi M, Magyarlaki T, Kovacs GL, Vermes I, Muehl D: **Activated platelet-derived microparticle numbers are elevated in patients with severe fungal (Candida albicans) sepsis.** *Ann Clin Biochem* 2012, **49**: 554-560.

Wu Q, Chen H, Fang J, Xie W, Hong M, Xia L: **Elevated Fas/FasL system and endothelial microparticles are involved in endothelial damage in acute graft-versus-host disease: a clinical analysis.** *Leuk Res* 2012, **36:** 275-280.

Xue S, Cai X, Li W, Zhang Z, Dong W, Hui G: **Elevated plasma endothelial microparticles in Alzheimer’s disease**. *Dement Geriatr Cogn Disord* 2012, **34**: 174-180.

Ye R, Ye C, Huang Y, Liu L, Wang S: **Circulating tissue factor positive microparticles in patients with acute recurrent deep venous thrombosis**. *Thromb Res* 2012, **130**: 253-258.

Zielinska M, Koniarek W, Goch JH, Cebula B, Tybura M, Robak T, Smolewski P: **Circulating endothelial microparticles in patients with acute myocardial infarction.** *Kardiol Pol* 2005, **62:** 531-542.
